# Dynamics of Brassinosteroid Response Modulated by Negative Regulator LIC in Rice

**DOI:** 10.1371/journal.pgen.1002686

**Published:** 2012-04-26

**Authors:** Cui Zhang, Yunyuan Xu, Siyi Guo, Jiaying Zhu, Qing Huan, Huanhuan Liu, Lei Wang, Guanzheng Luo, Xiujie Wang, Kang Chong

**Affiliations:** 1 Key Laboratory of Plant Molecular Physiology/Photosynthesis and Environmental Molecular Physiology, Institute of Botany, Chinese Academy of Sciences, Beijing, China; 2 Graduate University of the Chinese Academy of Sciences, Beijing, China; 3 Center for Molecular Systems Biology, Institute of Genetics and Developmental Biology, Chinese Academy of Sciences, Beijing, China; 4 National Plant Gene Research Center, Beijing, China; Peking University, China

## Abstract

Brassinosteroids (BRs) regulate rice plant architecture, including leaf bending, which affects grain yield. Although BR signaling has been investigated in *Arabidopsis thaliana*, the components negatively regulating this pathway are less well understood. Here, we demonstrate that *Oryza sativa* LEAF and TILLER ANGLE INCREASED CONTROLLER (LIC) acts as an antagonistic transcription factor of BRASSINAZOLE-RESISTANT 1 (BZR1) to attenuate the BR signaling pathway. The gain-of-function mutant *lic-1* and *LIC*–overexpressing lines showed erect leaves, similar to *BZR1*–depleted lines, which indicates the opposite roles of *LIC* and *BZR1* in regulating leaf bending. Quantitative PCR revealed *LIC* transcription rapidly induced by BR treatment. Image analysis and immunoblotting showed that upon BR treatment LIC proteins translocate from the cytoplasm to the nucleus in a phosphorylation-dependent fashion. Phosphorylation assay *in vitro* revealed LIC phosphorylated by GSK3–like kinases. For negative feedback, LIC bound to the core element CTCGC in the *BZR1* promoter on gel-shift and chromatin immunoprecipitation assay and repressed its transcription on transient transformation assay. LIC directly regulated target genes such as INCREASED LEAF INCLINATION 1 (*ILI1*) to oppose the action of BZR1. Repression of LIC in *ILI1* transcription in protoplasts was partially rescued by BZR1. Phenotypic analysis of the crossed lines depleted in both *LIC* and *BZR1* suggested that *BZR1* functionally depends on *LIC*. Molecular and physiology assays revealed that LIC plays a dominant role at high BR levels, whereas BZR1 is dominant at low levels. Thus, LIC regulates rice leaf bending as an antagonistic transcription factor of BZR1. The phenotypes of *lic-1* and *LIC*–overexpressing lines in erect leaves contribute to ideal plant architecture. Improving this phenotype may be a potential approach to molecular breeding for high yield in rice.

## Author Summary

Brassinosteroids (BRs) are phytohormones mediating multiple biological processes, such as development and stress response. They have been used in crops to produce high yield. In rice, the ideal plant architecture for high yield includes effective tillers, as well as height and leaf angle, which is modulated by BRs. Activation of BRI1–mediated BR signaling is well understood, but much less is known about its inactivating mechanism. Here, we found a gain-of-function mutant *lic-1* with the phenotype of the ideal rice plant architecture. The C3H-type transcription factor LIC antagonizes BZR1 to repress BR signaling in rice. We used BR to induce the negative regulator LIC and found that it functioned at high BR level, which may restrain plant development. LIC was phosphorylated by GSK3–like kinases. Phosphorylated LIC mainly localized in cytoplasm, whereas dephosphorylated LIC was in nucleus, which was regulated by BR treatment. LIC regulated transcription patterns of the downstream genes in an opposite direction to BZR1. BZR1 activated BR signaling, but the brake module of LIC repressed BR cascade amplification. LIC and BZR1 may balance BR signaling to control growth and development in rice.

## Introduction

Brassinosteroids (BRs) are plant steroid hormones that have been used to increase the yield of crops [1], [2]. BRs function in multiple developmental and physiological processes, including vascular differentiation, reproductive development, photomorphogenesis, and stress responses [3]–[5]. BR-deficient and -insensitive mutants show dwarfism, dark-green leaves, reduced fertility, and altered photomorphogenesis in the dark [6]–[9]. In rice (*Oryza sativa*), leaf-angle response to BRs is a specific physiological process. For example, the erect leaves of BR-deficient rice allow for greater growth density and higher grain yield [10]. Thus, analysis of genes involved in rice BR signaling could shed light on the molecular mechanisms of BR-regulated growth in monocots and help identify feasible approaches to increase rice yield by genetic engineering.

The BR signaling pathway has been well studied in *Arabidopsis*. Most of the signaling components of this pathway, from the BR receptor BRI1 and co-receptor BAK1 to nuclear transcription factors BZR1 and BES1/BZR2, have been identified [11], [12]. During the early events of BR signaling, BRI1 perceives BRs, thus inducing dissociation of the inhibitory protein BKI1, which results in association with and transphosphorylation of the co-receptor BAK1 [13]–[17]. BR signal kinases (BSKs) mediate signal transduction from BRI1 to BSU1 phosphatase through association with and phosphorylation of BSU1 [18]. BSU1 positively regulates BR signaling by dephosphorylating the negative regulator BR-insensitive 2 (BIN2). This process facilitates accumulation of unphosphorylated BZR1 and BES1/BZR2 in the nucleus [19]–[23], which directly or indirectly activate the expression of BR-responsive genes and regulate plant growth [21], [24], [25]. BZR1 is also responsible for the negative feedback of BR biosynthetic genes such as *CPD* by directly repressing transcription [26]. BZR1 and BES1 are major transcription factors in the BR signaling pathway [27]. BZR1 binds to the BR-responsive element (BRRE, CGTGT/CG) and mainly represses gene expression. BES1 binds to E-box by interacting with BIM1 or MYB30 to promote target gene expression [28]–[30]. BZR1 could also bind to E-box and BES1 to BRRE, so the functions of the family members may overlap [31], [32]. These are key transcription factors activating the BR signaling pathway in plants. Phosphatase 2A (PP2A) dephosphorylates BZR1 and also BRI1 in mediating BR signaling. BRI1 degradation depends on PP2A–mediated dephosphorylation that is specified by methylation of the phosphatase, thus leading to the termination of BR signaling [33]–[35]. However, how BR signals are repressed at the transcriptional level to elicit a “turn-off” pathway is less well known.

Rice, as a model monocot plant and one of the major crops, has been used to study the BR action mechanism. Both rice and *Arabidopsis* share primary BR biosynthesis and signal transduction pathways. A series of *Arabidopsis* orthologs of biosynthetic genes identified in rice include *D2*, *D11*, *BRD1*, *BRD2*, and *CPD* [36]–[38]. However, only a few members in the BR primary signaling pathway have been reported [39]–[41]. OsBRI1 and OsBAK1 are cell-surface receptor kinases that perceive BR signals. Os GLYCOGEN SYNTHASE KINASE 1 (OsGSK1), an ortholog of AtBIN2, is a negative regulator of rice BR signaling. Although the direct targets of BZR1 and BES1 have been identified in *Arabidopsis*, only a few targets have been identified in rice [31], [32]. The transcription factor OsBZR1, the closest ortholog of both BZR1 and BES1, has similar functions as its *Arabidopsis* orthologs [42]. OsBZR1 translocated from the cytoplasm to the nucleus in response to BR treatment in a process mediated by 14-3-3 proteins [42]–[44]. A pair of antagonizing HLH/bHLH factors, INCREASED LEAF INCLINATION (ILI1) and ILI1 BINDING bHLH (IBH1), function downstream of OsBZR1 to regulate cell elongation and lamina joint bending [45]. These studies suggest a conserved BR signaling mechanism in rice and *Arabidopsis*. BZR1 is a key component of the transcription pathway that activates BR signaling in both species. However, how to halt BR signaling at the node of transcription factors including BZR1 remains unclear.

A CCCH-type zinc finger protein, LEAF AND TILLER ANGLE INCREASED CONTROLLER (LIC) is involved in sterol homeostasis in rice [10]. Here, we studied the phenotypes of a rice *LIC* gain-of-function mutant *lic-1* and *LIC*-overexpressing rice lines to explore a novel mechanism of BR signaling. Both groups showed erect leaves and reduced BR sensitivity as compared with antisense lines. LIC was further characterized as an antagonistic transcription factor of BZR1 in regulating rice architecture. Furthermore, LIC is phosphorylated by GSK1/BIN2 (GSK3-like kinases), which affect translocation from the nucleus to cytoplasm. LIC may mediate a novel mechanism that represses the BR signaling pathway.

## Results

### *LIC* Truncation Results in Erect Leaves in Rice

**Figure 1:**
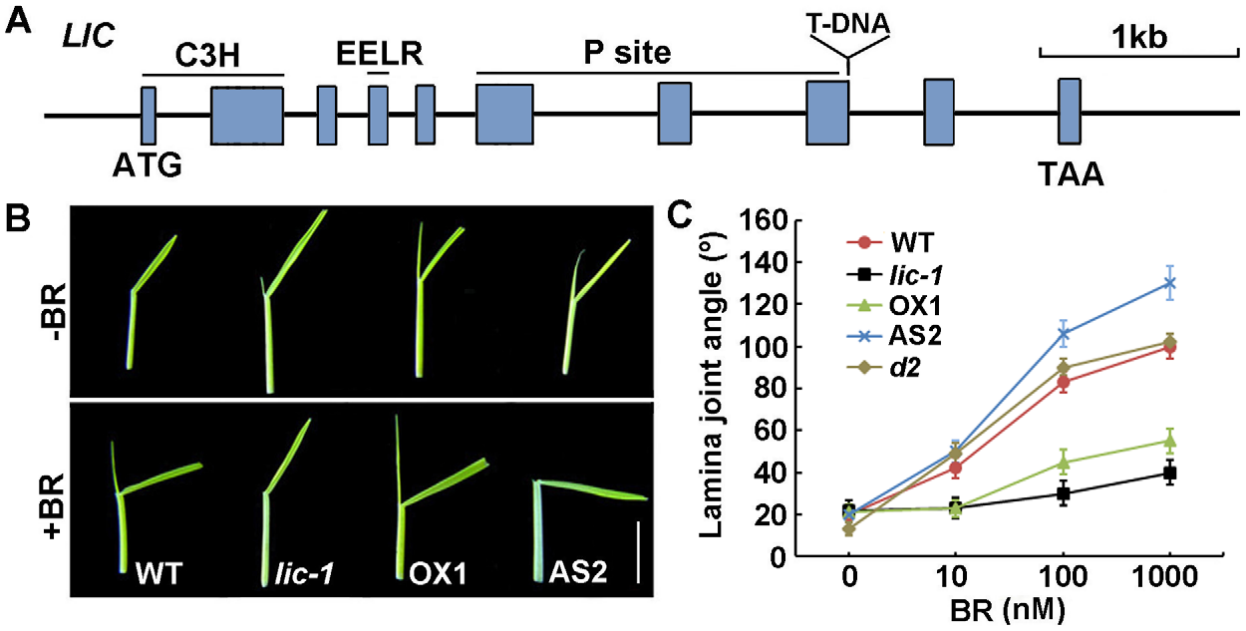
LIC negatively regulates BR signaling. (A) A diagram of the T-DNA insertion site in the *lic-1* mutant. LIC contains C3H, EELR and P site domains. The T-DNA is located near the P site domain. (B) Lamina joint assay of the *lic-1* mutant and the *LIC*-overexpressing lines in the presence of BR (the upper panel is treatment without BR and the bottom panel is 1 µM BR treatment; OX1, *LIC*-overexpressing line 1; AS2, *LIC* antisense line 2). Bar = 1 cm. (C) Quantification of lamina joint angle under different concentrations of BR. Lamina joint angles were averaged in 20 plants. BR-deficient mutant *d2* was a control. Data are mean±SD.

A T-DNA insertion line of *lic-1* was obtained from the Rice Mutant Database (http://rmd.ncpgr.cn) [46]. Molecular analysis revealed that the T-DNA was inserted in the eighth exon near the 3′ terminus of *LIC* and was predicted to cause the deletion of 110 amino acids (Figure 1A and Figure S1A). The inserted gene encodes a truncated LIC protein containing the CCCH DNA binding domain, EELR activation domain and the putative phosphorylation sites (Figure S1B and S1C).

Segregation analysis of the heterozygous *lic-1* with molecular evidence revealed an approximate 3∶1 (76/24) ratio of *lic-1* mutants to the wild-type, which indicates that *lic-1* is a dominant mutant. The crossed progenies of *lic-1* and the *LIC* antisense lines showed increased leaf angles that were similar to those of the antisense lines (Table S1). As compared with wild type, the *lic-1* line showed reduced leaf angles from tillering stage (Figure S2A and S2B). All *LIC*-overexpressing lines also showed erect leaves at tillering (Figure S2A, Figure S3, and Table S2). During the seedling stage, the wild type and *lic-1*, as well as overexpression lines, did not differ in leaf angle. Therefore, the phenotype of the *lic-1* mutant was consistent with the *LIC*-overexpression lines in terms of leaf angle.

BR biosynthetic genes *D2* and *D11* had repressed expression in antisense lines. In contrast, the expression of the biosynthetic gene *BRD1*, as well as the receptor gene *BRI1*, was enhanced in *lic-1* (Figure S4).

In rice, the physiologic processes of leaf bending and root growth are sensitive to BR [47], [48]. In the wild type (WT), increased leaf angle depended on the concentration of BR (Figure 1B and 1C). The overexpressing lines and *lic-1* showed reduced dependence on BR concentration in leaf bending. In contrast, the antisense line was more sensitive to BR dosage than the WT. In the antisense lines, the root growth patterns in response to 24-eBL (an active form of BR) were similar to leaf angle patterns (Figure S5A and S5B). Thus, *LIC* overexpression reduced the BR response, and *LIC* depletion caused hypersensitivity to BR in terms of leaf bending and root growth. Therefore, LIC may negatively regulate BR signaling in rice.

### *OsLIC* Is a Direct Target of OsBZR1

**Figure 2:**
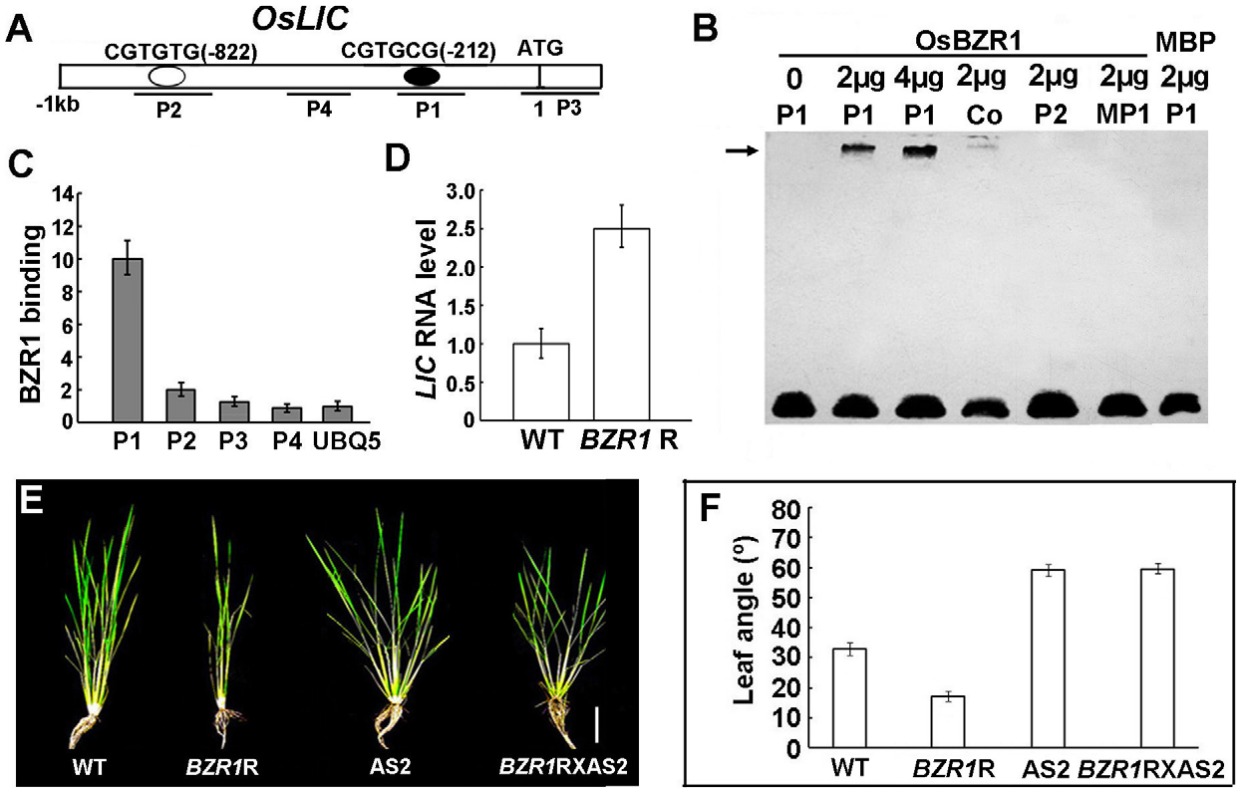
*LIC* promoter is targeted to the BZR1 protein. (A) A diagram of the *LIC* promoter containing BZR1 binding site (black circles): CGTGCG. White ring represents the sequence CGTGTG. Black lines P1–4 indicate the sequences tested in ChIP assays. P1 contained CGTGCG and P2 contained CGTGTG. But both elements were absent in P3 and P4. (B) Gel shift assay with BZR1 protein and the fragment sequences of the *LIC* promoter. The arrow indicates shifted bands caused by BZR1 binding to the *LIC* promoter P1 (CGTGCG). The unlabeled P1 was a competitive probe (Co). BZR1 could not bind to P2 (CGTGTG) or mutated P1 (MP1, CGAAAA ). MBP was a negative control. (C) ChIP assay revealed BZR1 enriched the *LIC* promoter fragment containing P1 *in vivo*. Data are mean ± SD (*n* = 3). *UBIQUITIN* promoter (*UBQ5*) was a negative control. (D) Increased expression pattern of *LIC* in the RNAi line of *BZR1* (*BZR1*R). Data are mean ± SD (*n* = 3). (E) Phenotypes of the progeny of *BZR1*R X AS2 and the parent lines *BZR1*R and AS2, as well as the wild type. The plants analyzed in this experiment were 30 days old. Bar = 20 cm. (F) Quantification of the leaf angles of the progeny *BZR1*R X AS2 and the parent lines *BZR1*R and AS2, as well as the wild-type in (E). Leaf angles were averaged in 15 plants. Data are mean ± SE.

Bioinformatics analysis revealed the BZR1 binding site BRRE (CGTGT/CG) [26] present in the promoter of *LIC* (Figure 2A). EMSA was used to examine BZR1 binding to the cis-elements in the *LIC* promoter *in vitro*. When the purified BZR1 protein was incubated with the reaction mixture, a shifted band appeared in the upper part of the gel but not in the control MBP. The greater the amount of BZR1 in the incubation, the greater the amount of shifted band on the gel. When the competitive unlabeled probe (Co) was added to the system, the shifted band was suppressed. In contrast, neither mutated P1 (MP1, CGAAAA ) nor P2 (CGTGTG) shifted under the same conditions (Figure 2B). We performed chromatin immunoprecipitation (ChIP) assay with WT rice (Figure S9). Real-time PCR revealed a fragment of the *LIC* promoter containing the P1 binding element significantly enriched as compared with the reference gene promoter (*UBQ5*) and control fragments (P2, P3 and P4; Figure 2C). In the RNAi lines of *BZR1*, *LIC* transcription was increased (Figure 2D). Thus, BZR1 binds to the *cis*-element in the *LIC* promoter, and knockdown of *BZR1* leads to upregulation of *LIC*.

We crossed the *BZR1* RNAi lines with erect leaves to the *LIC* antisense lines with increased leaf bending to explore the genetic relationship of the lines. By molecular identification (Figure S6A and S6B), phenotypic analysis revealed an increased leaf bending phenotype in the progenies, which was similar to that of the *LIC* antisense lines (Figure 2E and 2F). Therefore, *BZR1* may functionally depend on *LIC* in terms of genetics.

### LIC Is a Substrate of GSK3–Like Kinases, the Rice Orthologs of AtBIN2

**Figure 3:**
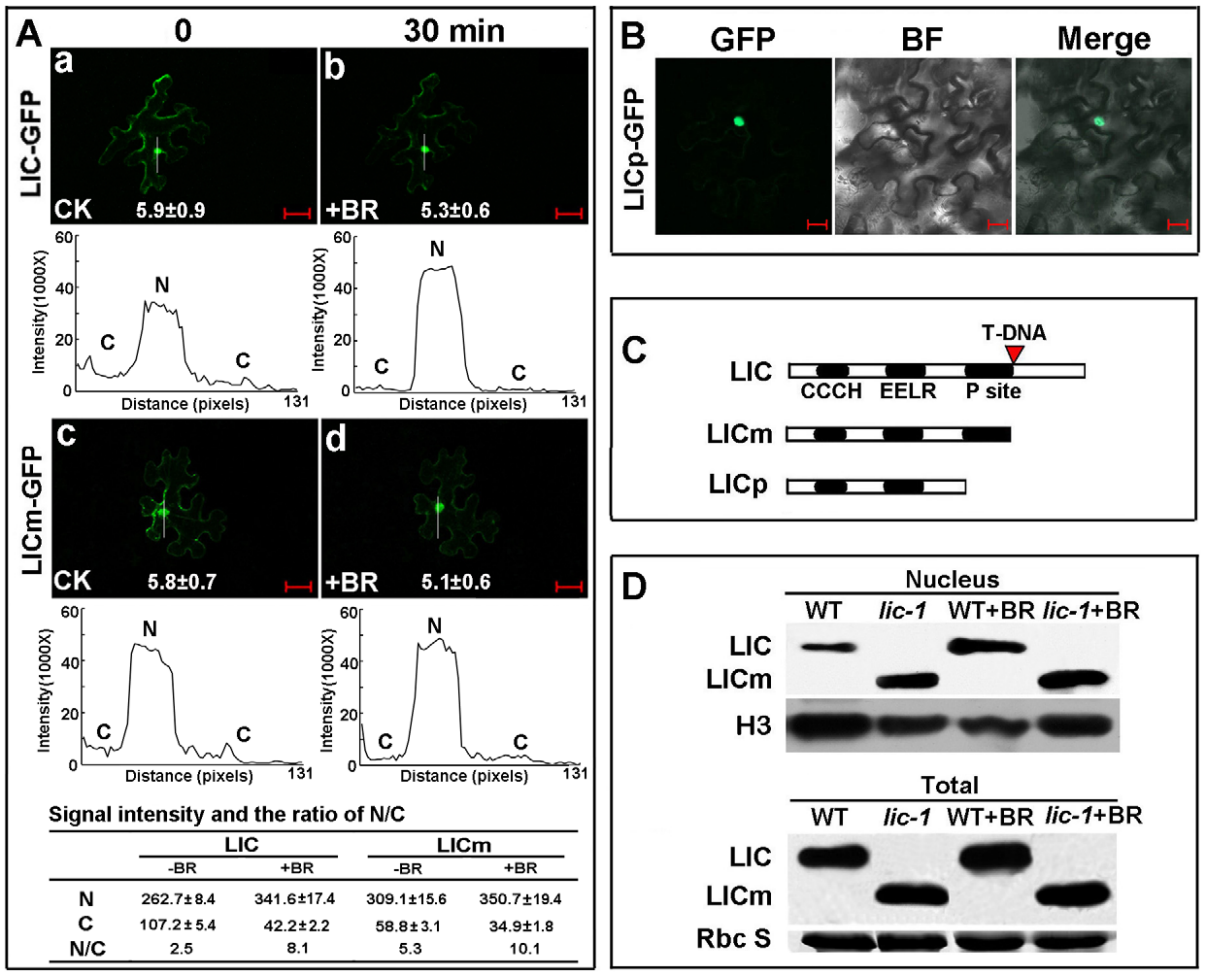
LIC accumulates in the nucleus in response to BR treatment. (A) LIC accumulated in the nucleus in response to BR induction: (a) and (b) LIC-GFP fusion protein localized in both the nucleus and cytoplasm; LIC-GFP fluorescence intensity was weakened in the cytoplasm and enhanced in the nucleus with 1 µM 24-eBL treatment for 30 min. (c) and (d) LICm (mimic of *lic-1*) accumulated in the nucleus after 1 µM 24-eBL treatment similar to the intact LIC protein pattern. Numbers in each image show the mean signal of the total cell (1000×) and standard errors calculated from 10 cells for each treatment. The white lines inside the images show the areas used for line scan measurements that yielded plot profiles shown in the lower panels. The table shows signal intensities (10^5^×) and the ratios between nuclear and cytoplasmic (N/C) from represented areas. N, nuclear signal; C, cytoplasmic signal. The scale bar is 20 µm. (B) LICp-GFP fusion protein (deletion of both the C-terminus and P site) localized in only the nucleus. Bars = 20 µm. (C) A diagram for LIC protein (containing CCCH domain, EELR, P site and C terminus), LICm (deletion of the C-terminus, mimic of *lic-1*) and LICp (deletion of both the C-terminus and phosphorylation sites). (D) Immunoblotting analysis of LIC and LICm protein levels in the nuclear fractions and total protein. LICm localization was more in the nucleus, which is similar to wild-type LIC in BR-treated (1 µM) plants. LIC levels in the total protein did not change under the same condition. Histone 3 was the loading control for the nuclear fraction and Rubisco small subunit was the loading control for total protein.

Transformed LIC-GFP fusion protein was used to investigate subcellular localization. With BR treatment, GFP-tagged LIC was rapidly weakened in the cytoplasm within 30 min but was enhanced in the nucleus (Figure 3A a and b). The ratio of GFP-tagged LIC in the nucleus to that in the cytoplasm (N/C ratio) was significantly increased with BR treatment. Although LICm, mimicking the C-terminus-truncated protein, was distributed in the nucleus and cytoplasm, the cytoplasmic signal of LICm was clearly weaker than that of intact LIC (Figure 3A c and d; 3C). Digital signal assay demonstrated a lower ratio of LIC than truncated LICm in the nucleus. The LICm pattern showed a similar increased N/C ratio in response to BR treatment. In contrast, the truncated protein LICp, lacking the putative phosphorylation sites (designated P site in Figure 1A and Figure 3C) was localized only in the nucleus (Figure 3B). Western blot analysis revealed a greater LIC band in the nucleus of *lic-1* as compared with the WT. Intensity of the nuclear band was enhanced by treatment with 24-eBL (1 µM) for both *lic-1* and the WT. At the total protein level, the signal intensity of LIC in the WT and *lic-1* was not significantly different after treatment (Figure 3D). Thus, the translocation of LIC from the cytoplasm to the nucleus may be regulated by BR treatment and depend on the phosphorylation status of LIC.

The GSK3-like kinase BIN2 phosphorylates BZR1 through the conserved GSK3 kinase phosphorylation sites (S/TxxxS/T) and promotes its cytoplasmic retention in *Arabidopsis* [49]. In rice, whole-genome screening analysis revealed two putative orthologs of BIN2, OsGSK1 and OsSKETHA [43].

**Figure 4:**
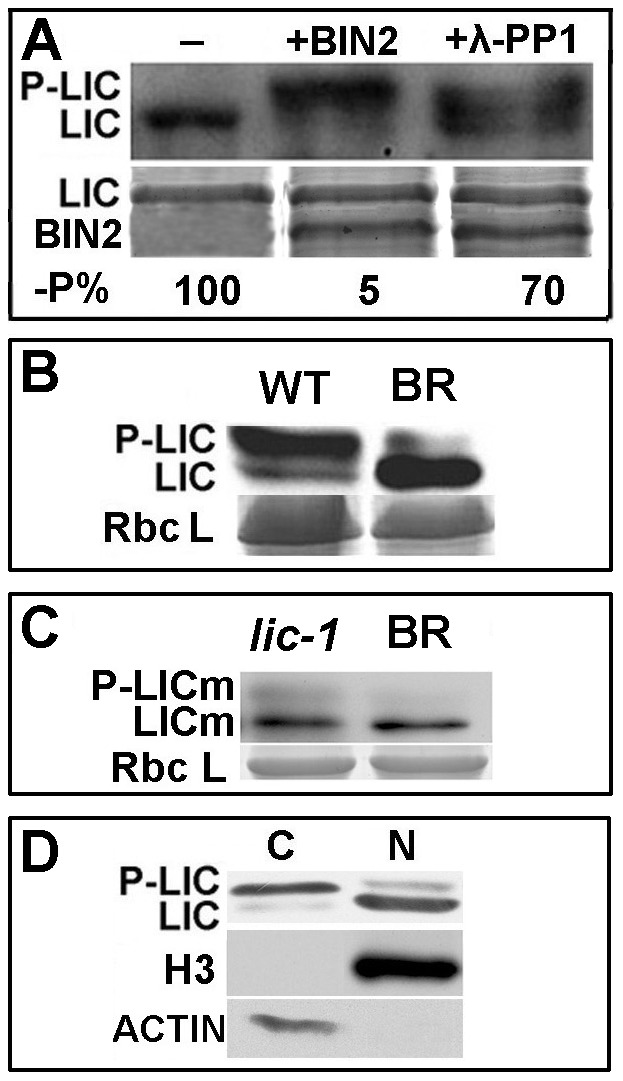
LIC is phosphorylated by BIN2/GSK1. (A) Immunoblotting analysis to demonstrate that LIC was phosphorylated by BIN2. LIC phosphorylation was antagonized by λ-phosphatase 1 (PP1). The phosphorylation status of LIC is illustrated by autoradiography of an anti-LIC antibody in the top panel. The amount of protein is shown with Coomassie Blue staining in the bottom panel. The levels of unphosphorylated LIC relative to the control without BIN2 and PP1 (-P%) were calculated after normalization against the intensity of Coomassie Blue staining, and these values are shown beneath the gel images. (B) Treatment with BR (1 µM) decreased the levels of phosphorylated LIC and increased that of unphosphorylated LIC. Rice plants were grown for 2 weeks and then soaked with 1 µM 24-eBL (+) or mock solution (−) for 3 h. LIC protein was analyzed by immunoblotting with an anti-LIC antibody (upper panel). The loading control with Coomassie Blue staining is shown in the bottom panel. (C) The mutated protein LICm caused decreased phosphorylation in the *lic-1* mutant. The 24-eBL concentration was 1 µM. (D) Immunoblotting assay for LIC protein in the nuclear and cytoplasmic fractions. Dephosphorylated LIC was dominant in the nucleus (N), and phosphorylated forms were dominant in the cytoplasm. Nuclear and cytoplasmic protein fractions were extracted from 2-week-old rice seedlings. Histone 3 was a marker for the nuclear protein and ß-actin for the cytoplasmic protein.

Yeast two-hybrid assay revealed that LIC but not forms LICm and LICp interact with GSK1 and SKETHA, as well as AtBIN2. The mutated form did not interact with them (Figure S7). Western blot analysis revealed that LIC was two bands and the larger one was enhanced by incubation with BIN2. Furthermore, the intensity of the larger band was reduced by the addition of λ-phosphatase 1 (Figure 4A), which agreed with the prediction that 5 typical phosphorylation sites of GSK3-like kinases (S/TxxxS/T) were deposited in the P site domain of LIC protein.

When plants were treated with 24-eBL (1 µM), the phosphorylated form of LIC was suppressed (Figure 4B), whereas the dephosphorylated form was increased. The P-LICm was weaker than that for LICm (Figure 4C). Western blot revealed dephosphorylated LIC accumulated in the nuclear fraction, with the phosphorylated form mainly in the cytoplasm (Figure 4D).

**Figure 5:**
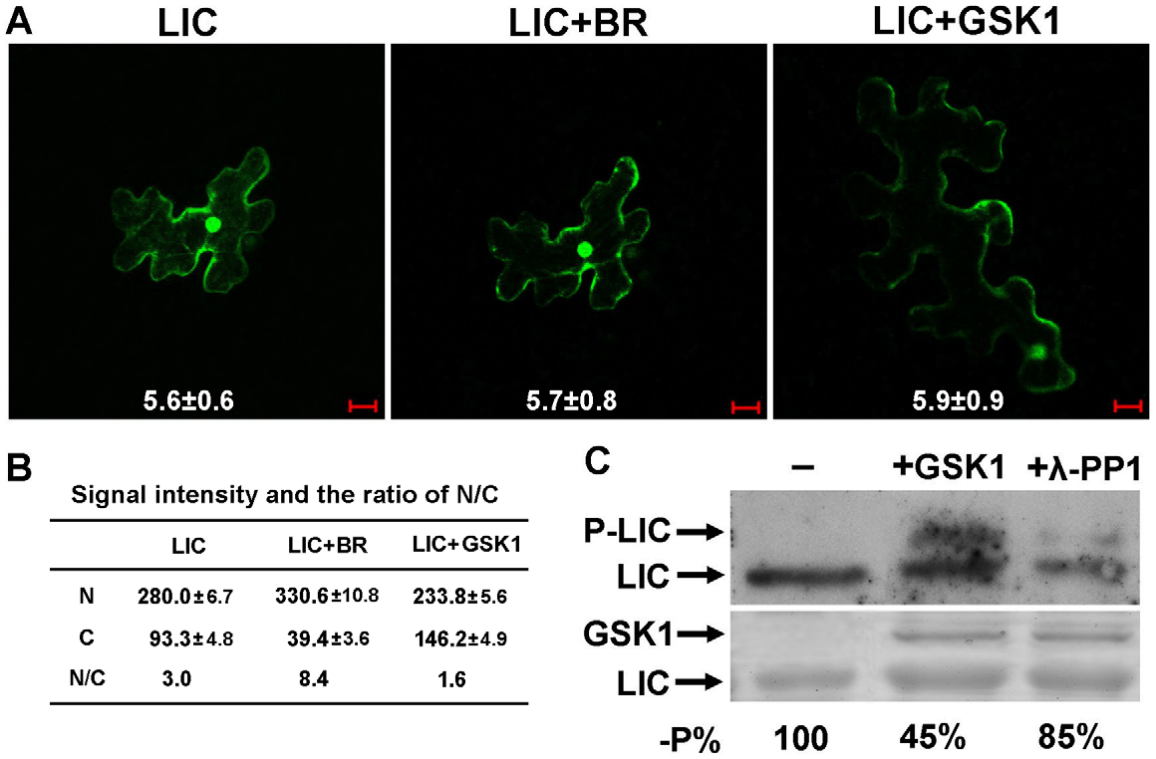
GSK1 phosphorylates LIC and reduces its nuclear localization. (A) The LIC-GFP fusion protein localized in both the nucleus and the cytoplasm (left). LIC-GFP fluorescence intensity was enhanced in the nucleus and weakened in the cytoplasm after treatment with 1 µM 24-eBL (middle). LIC-GFP fluorescence intensity was weakened in the nucleus when co-transformed with GSK1 (right). Numbers in each image show the mean signal intensity (1000×) from at least 10 cells. Data are mean±SE. Bars = 20 µm. (B) Quantification of the fluorescence intensity (10^5^×) and the ratio between the nucleus and the cytoplasm (N/C) in represented areas. N, nuclear signal; C, cytoplasmic signal. (C) Immunoblotting to demonstrate the phosphorylation of LIC by GSK1, which was antagonized by λ-phosphatase 1 (PP1). The level of phosphorylation is shown by autoradiography with an anti-LIC antibody in the top panel and the loaded amount of proteins is indicated by Coomassie Blue staining in the bottom panel. The levels of unphosphorylated LIC relative to the control without GSK1 and PP1 (-P%) were calculated after normalization against the intensity of Coomassie Blue staining and these values are shown beneath the gel images.

Transformed cells with *GFP*-tagged *LIC* showed the N/C ratio of LIC with digital fluorescence signals was 3.0 (Figure 5A and 5B). The nuclear distribution of LIC was enhanced with 24-eBL treatment. In contrast, in cells co-transformed with both *GSK1* and *LIC*, less of LIC localized in the nucleus. Western blot analysis demonstrated that phosphorylated LIC was upregulated after incubation with GSK1 but downregulated with λ-phosphatase 1 (Figure 5C). This result suggested that GSK1 phosphorylated LIC, which might repress its localization in the nucleus.

### LIC Regulates BZR1 in a Negative Feedback Loop

**Figure 6:**
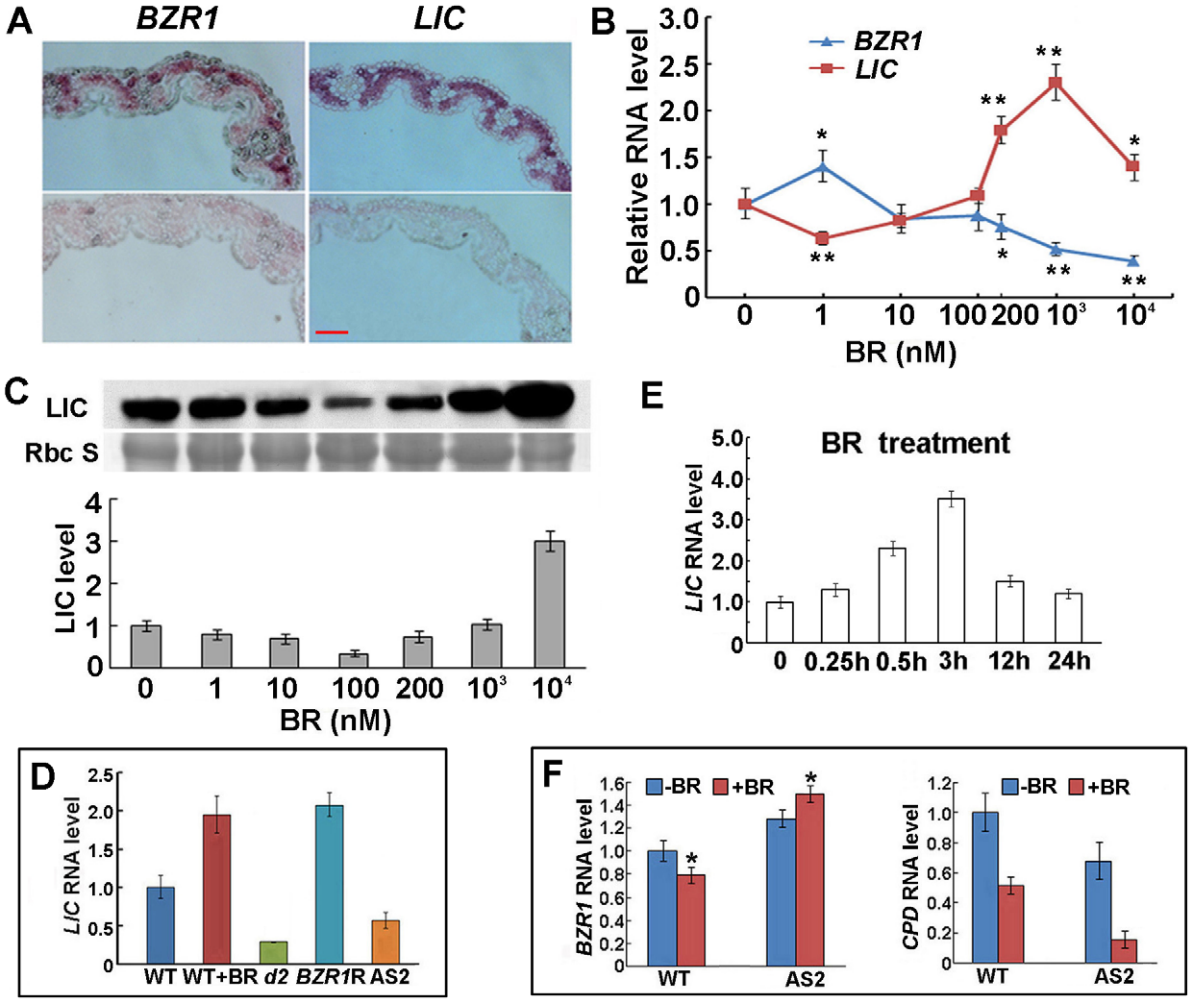
LIC and BZR1 expression patterns and their responses to BR. (A) RNA *in situ* expression of *LIC* and *BZR1* on the abaxial and adaxial sides of leaves (the bottom panel represents the negative control with sense probes). Bar = 10 µm. (B) *LIC* and *BZR1* transcriptional expression response to various concentrations of BR. Data are mean ± SD (*n* = 5). *P<0.05 and **P<0.01 compared with no BR treatment as determined by Student's *t* test. (C) Immunoblotting to show the response of LIC protein expression to BR. LIC was repressed by low concentrations of BR (<100 nM) and induced by high concentrations of BR (>200 nM). Coomassie Blue staining served as the loading control. The levels of LIC were calculated after normalization against the intensity of Coomassie Blue staining in 3 replicated experiments, and the quantified values are shown beneath the gel images. Data are mean ± SE. (D) *LIC* transcriptional expression with BR treatment in wild-type (WT) and BR-deficient mutant *d2* and *BZR1* RNAi transgenic lines (*BZR1*R). *LIC* antisense line 2 (AS2) was a control. Data are mean ± SD (*n* = 3). (E) Time course response of transcription expression of *LIC* to BR (1 µM). *LIC* was rapidly induced by BR. Data are mean ± SD (*n* = 3). (F) *BZR1* and *CPD* transcriptional response to BR treatment in the wild type and *LIC a*ntisense lines. For *BZR1*, data are mean ± SD (*n* = 5). *P<0.05, compared with no BR treatment. For *CPD*, Data are mean ± SD (*n* = 3).

Expression pattern assay demonstrated *BZR1* and *LIC* with overlapping and distinct expression patterns in different organs (Figure S10). The expression of *LIC* was distributed from the abaxial to adaxial sides in leaves. In contrast, the expression of *BZR1* was dominant in the abaxial sides of leaves (Figure 6A).

The effect of BR on *LIC* transcription showed repression at low (1 nM) and activation at high (>100 nM; Figure 6B) 24-eBL concentrations. This matches the phenotype of root growth (Figure S5C). The peak of *LIC* transcription occurred with 1 µM 24-eBL. In contrast, the mRNA level of *BZR1* was increased with low levels of 24-eBL (1 nM) and decreased with high levels (>100 nM; Figure 6B). Western blot analysis demonstrated that the pattern of LIC protein level was similar to the mRNA pattern with low levels of 24-eBL. However, the reduced negative-peak occurred with a higher 24-eBL concentration (100 nM) than for the RNA (1 nM). Additionally, the increased protein expression was sustained with up to 10 µM 24-eBL (Figure 6C). Thus, LIC expression may be downregulated by a low level of BR but upregulated by higher concentrations.

In the mutant *d2*, BR deficiency caused *LIC* expression reduced to only 20% the WT level. *BZR1* depletion resulted in increased *LIC* expression (Figure 6D). Time-course assay revealed *LIC* expression gradually increased from 15 min up to 3 h during BR treatment (1 µM 24-eBL) (Figure 6E). In the *LIC* antisense lines, *BZR1* expression was enhanced with the treatment, which was opposite to that in the WT. Transcription expression of the BZR1 target gene *CPD* was greatly repressed by BR treatment in the antisense lines (Figure 6F). Thus, LIC may be involved in the negative regulation of *BZR1*.

To screen LIC potential target motifs, genes with altered expression of the *LIC* antisense lines in microarray data were re-sorted. In previous microarray analysis [10], the expression of 1,175 genes was altered by at least 2-fold in the *LIC* antisense lines. We extracted 1 kb of upstream sequences of the genes with altered expression patterns as the predicted promoters and then used MEME (http://meme.sdsc.edu/meme/cgi-bin/meme.cgi) to locate the recurrent motifs. We extracted 14 motifs representing the potential regulatory cis-elements from the altered genes (Figure S8A). EMSA results suggested that 3 elements (S1–3) containing a core sequence TCGC bound to LIC (Figure S8B). Therefore, the core sequence TCGC is one of the LIC-binding elements.

**Figure 7:**
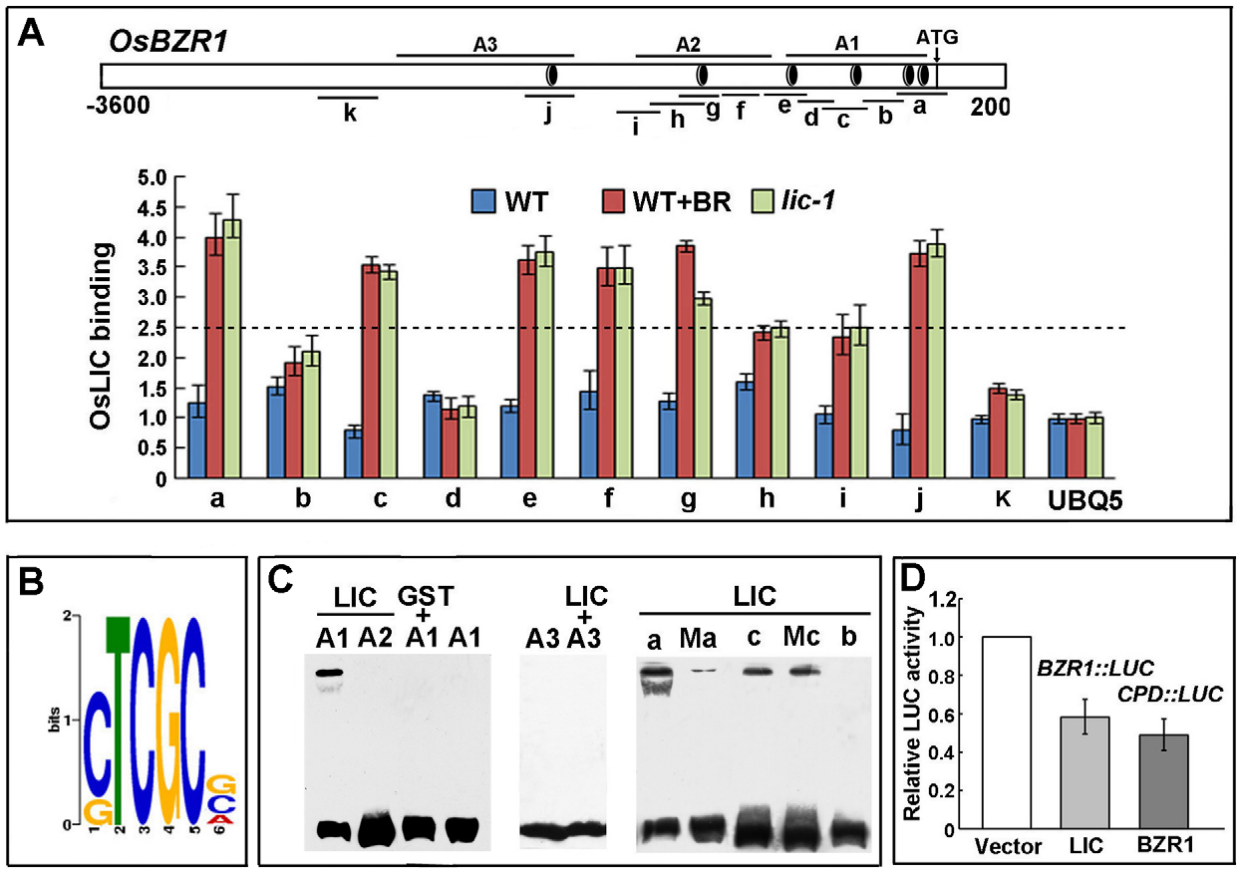
LIC binds to *BZR1* and represses its transcriptional expression. (A) ChIP assay to illustrate LIC binding to the *BZR1* promoter. The binding was enhanced in the *lic-1* mutant and in wild-type plants treated with BR. The black circles with a white ring indicate the putative binding motif S (CTCGC). A1, A2 and A3, the probes used in EMSA; a–k, sequences tested in ChIP assay; a–c, also as sub-sequences of A1 used in EMSA. The *UBQUITIN5* promoter was a control. (B) Putative binding motif S predicted by MEME software (http://meme.sdsc.edu/meme/cgi-bin/meme.cgi). Sequence logo shows the frequencies relative to the information content at each position. (C) Gel shift assay to illustrate LIC binding to the putative core binding sequence. LIC bound to the *BZR1* promoter A1 fragment (4 elements) but not to the A2 or A3 fragments (one element); GST could not bind to A1. The right panel shows LIC binding to sub-sequences of the A1 fragment a–c, Ma and Mc (CTCGC were mutated to AAAAA ). (D) Transient transfection assay indicating that LIC inhibits *BZR1pro:LUC* reporter gene expression in *Arabidopsis* protoplasts. The *AtCPDpro:LUC* reporter gene repressed by BZR1 was the control. Data are mean±SD of triplicate experiments.

The core sequence was deposited in *BZR1* gene. ChIP data revealed that LIC bound to the *BZR1* promoter in various regions, such as, a, c, e, f, g and j, which were upregulated by BR treatment in the WT (Figure 7A and Figure S9). In contrast, the remaining fragments, which lacked TCGC, such as, b, d, h, I and k, had lower binding affinities. In the *lic-1* mutant, the bound patterns were similar to that of the BR-treated WT.

We used MEME to locate the recurrent motifs among the multiple region sequences identified on ChIP. The motif CTCGC (denoted as S, containing the TCGC core sequence) was consistently found with high values (Figure 7B and Figure S8C). EMSA demonstrated that LIC bound specifically to CTCGC (Figure S8D). Mutated probes M1 ( ATCGCG) and M2 (CTCGCT ) led to decreased intensity of the shifted band. In contrast, mutated M3 (CAAAAG) caused the band to disappear.

The fragments with multiple copies of the element on the *BZR1* promoter were used to further confirm the binding activity. EMSA results suggested that binding affinities of LIC were related to copy numbers of the elements in the *BZR1* promoter (Figure 7C). Transient transfection assay revealed that LIC protein repressed the expression of *BZR1pro:LUC* in *Arabidopsis* protoplasts as compared with the control (vector; Figure 7D) [26]. Therefore, LIC may be a primary transcription factor targeting *OsBZR1* to regulate the BR signaling pathway.

### LIC and BZR1 Function Antagonistically in Regulating Downstream Genes

**Figure 8:**
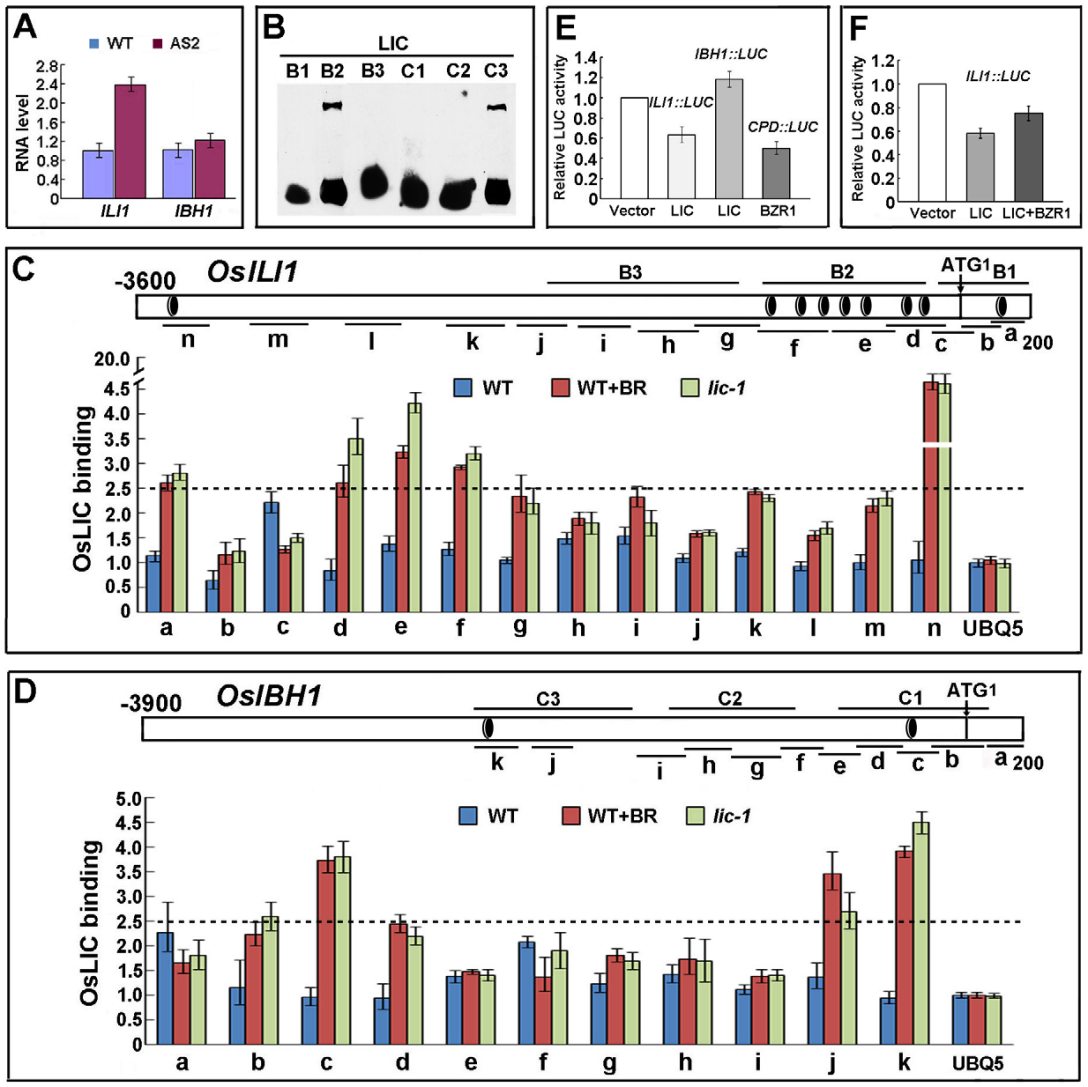
Opposite regulation of downstream genes in BR signaling by LIC and BZR1. (A) Transcriptional expression patterns of *ILI1* and *IBH1* in the *LIC* antisense line (AS2). Data are mean ± SD (*n* = 3). (B) Gel shift assay to illustrate LIC binding to the different fragments of the *ILI1* and *IBH1* promoters. *ILI1* B2 and *IBH1* C3 contain the binding element S. *ILI1* B1, B3, *IBH1* C1 or C2 fragments contain no or less binding elements. (C) and (D) ChIP analysis of LIC binding to the *ILI1* and *IBH1* promoters by use of anti-LIC antibody. The binding was enhanced in the *lic-1* mutant and in wild-type plants in the presence of BR. The black circle with white ring indicates the binding element S. B1–3 and C1–3 are the probes used in (B), and a–n (used in (C)) and a–k (used in (D)) indicate the sequences tested in ChIP assay. The *UBQUITIN5* promoter was used as a control. (E) Transient transfection assay to illustrate that LIC repressed *ILI1pro:LUC* and activated *IBH1pro:LUC* reporter gene expression in *Arabidopsis* protoplasts (the 403-bp *ILI1* promoter indicated as B2 in (C) and the 451-bp *IBH1* promoter indicated as C3 in (D) were used). The inhibition of *AtCPDpro:LUC* reporter gene expression by BZR1 was the control. Data are mean± SD. (F) Transient transfection assay indicated that LIC and BZR1 antagonistically regulate *ILI1pro:LUC* reporter gene expression. Data are mean ± SD.

To determine the potential antagonistic functions of both genes, we analyzed the expression patterns of their potential downstream genes. BZR1 mainly binds IBH1 to affect the balance of a pair of antagonistic HLH/bHLH transcription factors ILI1 and IBH1 in rice [45]. In an *LIC*-depleted line (AS2), *ILI1* expression was higher than in the WT, whereas *IBH1* transcription was not significantly altered (Figure 8A). The core motif sequence CTCGC of LIC target was present as a glomerate pattern in *ILI1* but as a sparse pattern in *IBH1*. EMSA data indicated that the fragment containing the sequence B2 in *ILI1* strongly bound to LIC. In contrast, the signal of C3 in *IBH1* with a single core element was weaker (Figure 8B). ChIP analysis of the potential target *ILI1* after BR treatment in the WT demonstrated significant changes (>2.5-fold) in binding in diverse regions such as a, d, e, f and n, but not in regions such as b, c, h, i, j and l (Figure 8C). *IBH1* exhibited a similar pattern as *ILI1* on ChIP analysis, but the copy number of the core element on the *IBH1* fragments, such as c and k, was much lower than that for *ILI1* (Figure 8D). Unexpectedly, the change appeared in the region without the core motif such as j, so other unknown motifs may be involved. To further explore the potential activity of the transcription factor with its targets *ILI1* and *IBH1*, we used a protoplast transfection assay. LIC repressed the expression of *ILI1pro:LUC* but activated that of *IBH1pro:LUC* (Figure 8E). Competitive binding assay showed that the repression activity of LIC on *ILI1* was weakened by co-expression of *BZR1* (Figure 8F). Thus, LIC dominantly repressed *ILI1* expression and weakly bound to *IBH1* to enhance expression to balance the regulation activity of BZR1.

## Discussion

**Figure 9:**
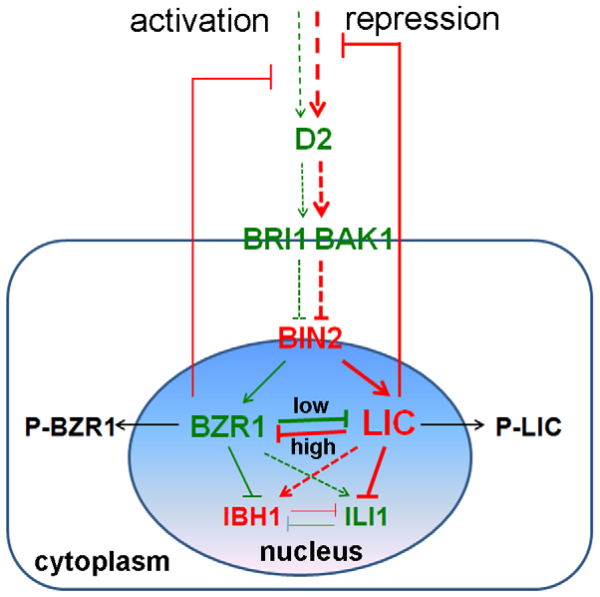
A hypothetical working model for the role of LIC in the BR signaling pathway. BZR1 is a positive transcription factor and represents an activation pathway, whereas LIC functions antagonistically as a negative transcription factor and mediates a “brake” pathway in BR signaling. Both BZR1 and LIC are phosphorylated by BIN2/GSK1 and transported to the cytoplasm in the absence of BR (green represents positive members and the activation pathway, and red represents negative members and repression pathway in BR signaling).

BZR1 is one of the key nodes for components in the BR signaling pathway. Modification of phosphorylation on BZR1 modulates BR signaling to mediate growth and development in *Arabidopsis* [33], [34]. In this study, we identified LIC as a negative regulator of BZR1 to halt BR signaling to control leaf angle in rice. LIC antagonizes BZR1 by repressing its transcription in leaf bending (Figure 9). Like BZR1, the transcription factor LIC is phosphorylated by GSK1/BIN2. LIC and BZR1 are a pair of antagonistic transcription factors that repress each other during transcription. However, their repression strength may depend on BR level. The gain-of-function mutant *lic-1* showed that LIC repressed *BZR1* transcription and leaf bending, which may mimic a BR signaling balance to switch on the “brake.” The transcriptional expression of *LIC* and its protein accumulation in the nucleus were induced by BR. LIC dominantly binds to *BZR1* and its target *ILI1* and weakly to *IBH1*. In contrast, BZR1 mainly targets *IBH1* to affect the balance of a pair of antagonistic HLH/bHLH transcription factors, except to bind *LIC*. The “seesaw” mechanism of the antagonistic function may work at various BR levels during plant development. BZR1 may function at a low level to promote signaling and LIC at higher levels for braking. Our data suggest that LIC is a component of the BR signaling pathway and mediates a novel braking module that represses BR signaling to control plant development.

### LIC Is a Major Negative Regulator Mediating Signaling from GSK3 Kinases

Leaf bending is a specific phenotypic response to BR in rice [47]. *LIC*-depleted rice lines show increased leaf bending, which mimic the phenotypes of enhanced responses to BR, such as the OsBAK1-overexpressing lines [40]. Consistently, the gain-of-function mutant *lic-1* and overexpressing lines show erect leaves similar to OsBZR1- and OsBAK1-depleted lines. Reverse expression patterns of downstream genes such as *ILI1* and *IBHI1* were found in the silenced lines of *BZR1* and *LIC*. Similarly like BZR1, LIC acts in an early BR response, because its expression was induced by BR treatment within 15 min. BR-induced LIC accumulation in the nucleus was a rapid response to BR and acts as an upstream component in BR signaling. Therefore, *LIC* functions negatively in the BR-mediated regulation of leaf bending.

LIC, with phosphorylation sites of GSK1/BIN2 kinases, interacts with BIN2 and its rice orthologs GSK1 and SKETHA and could be phosphorylated. The C-terminus-truncated LICm, as well as P-site-truncated LICp, show decreased interaction with GSK1/BIN2 and consequently display lower phosphorylation levels and greater accumulation in the nucleus to constitutively regulate downstream genes. The shuttle of LIC between the nucleus and cytoplasm was regulated by BR treatment and might depend on the phosphorylation status of LIC. This shuttle localization pattern depended on BIN2/GSK1, as seen with BZR1/BES1 [23], [42], [50]. Our results suggest that LIC directly mediates BR signaling from GSK3 kinases.

### LIC Antagonizes BZR1 in Rice Leaf Bending

BZR1 is a key component positively regulating BR signaling, whereas LIC plays a negative role in the signaling pathway. With BR treatment, LIC and BZR1 accumulate in the nucleus and regulate downstream genes. BZR1 represses BR-downregulated genes through the downregulation of BIN2 phosphorylation and decreases cytoplasmic retention mediated by 14-3-3 proteins during BR-mediated induction [42], [50]. BZR1 and BES1 gain-of-function mutants in *Arabidopsis* are hypersensitive to BR. Depleted AtBES1 leads to reduced BR sensitivity [51]. The RNAi lines of OsBZR1 are insensitive to BR and show erect leaves [42]. Our genetic analysis and molecular data suggested that *LIC* and *BZR1* work on rice leaf bending in a genetic pathway, but their roles are opposite to each other. EMSA and ChIP data, as well as transcription assay data, indicated that the BZR1 protein directly represses *LIC* expression via the specific BRRE motif (CGTGCG). We found that LIC protein recognizes the *BZR1* gene through the core element (CTCGC) to repress its transcription. The expression of both genes may be induced by BR treatment at various concentrations. BR treatment at low concentrations (10^−9^ M) induced the expression of *BZR1* and promoted the dephosphorylation of BZR1 protein as an activation mechanism. However, BR treatment at high concentrations (up to 10^−7^ M) induced *LIC* expression. The repression of *BZR1* transcriptional expression by LIC is enhanced by high BR levels. Therefore, LIC and BZR1 antagonize each other in controlling BR-mediated leaf bending.

LIC, with only one CCCH domain, binds DNA or RNA *in vitro* (Figure S11) [10]. It prefers to recognize the core sequence CTCGC, which is present in genes such as *BZR1 and ILI1*, to regulate BR signaling in rice. Our finding of the motif binding to LIC with specificity will provide new insights into this family.

BZR1 is a transcription factor that represses the expression of downstream genes such as *OsIBH1*, which is responsible for leaf bending. ILI1/PRE1 and IBH1 promote or repress cell elongation downstream of BZR1 in rice and *Arabidopsis* [45], [52]. Overexpression of *ILI1* causes increased leaf bending, whereas overexpression of *IBH1* results in erect leaves in rice. EMSA and ChIP results suggested that LIC greatly represses *ILI1*, the positive partner of *OsIBH1*. As well, LIC weakly binds to *OsIBH1* promoter to enhance its transcriptional expression. This pattern is similar to BZR1 weakly binding to the promoter of *ILI1*, which is induced by BR [45]. In regulating downstream genes, LIC may play a major role in repressing positive regulators such as ILI1, and BZR1 may function to repress negative regulators such as IBH1. Therefore, a novel negative regulation module of BR signaling is parallel to and antagonizes the BZR1 signaling pathway to regulate leaf bending. In plant development, *LIC* and *BZR1* show various spatial and temporal expression patterns. BZR1 acts in the presence of low levels of BR, whereas LIC is predominantly activated by high levels of BR and antagonizes BZR1 to prevent intense activation of the BR cascade. The novel negative regulation module of LIC and the positive one of BZR1 in mediating leaf bending may help in designing ideal plant architecture for improving photosynthesis efficiency during rice development. The approach may have potential in rice molecular breeding for high yield.

## Materials and Methods

### Plant Materials and Growth Conditions

Rice (*Oryza sativa* ssp. *japonica* var. Zhonghua 10) plants were grown in the field or in the greenhouse at 30°C/25°C (day/night) cycles. For the analysis of BR induction in leaf bending and root growth, rice seeds were sterilized with 1% NaClO and grown in half-strength Murashige and Skoog (MS) medium with the indicated concentrations of 24-eBL (Sigma-Aldrich, St. Louis, MO, USA) at 30°C under continuous light. Seedlings were examined 7 days after germination. For every transgenic rice plant, 3 lines were used.

### Leaf-Bending Assay

Sterilized seeds were grown for 8 days in a dark chamber. Uniform seedlings were then sampled by excising segments of approximately 2 cm that contained the second-leaf lamina joint under dim light conditions. These were floated on distilled water containing various concentrations of 24-eBL. After incubation in a dark chamber at 30°C for 72 h, the angle between the lamina and the sheath was measured [47].

### Total RNA Isolation and Quantitative RT–PCR Analysis

Total RNA was extracted from 2-week-old seedlings by using the Trizol RNA extraction kit (Invitrogen, Carlsbad, CA, USA). The first-strand cDNAs were synthesized by use of M_MLV reverse transcriptase (Promega) and used as RT-PCR templates. Quantitative real-time PCR analysis involved an Mx3000P (Stratagene) with a SYBR green detection protocol. RT-PCR was repeated at least 3 times for each harvested samples with gene-specific primers and *ACTIN1* as the reference gene (see Table S1). The data were analyzed by the *C_T_* formula considering amplification efficiencies for every PCR [53].

### Vector Construction and Plant Transformation

The cDNA of LIC from a rice cDNA library was amplified by PCR and ligated into pUN1301 binary vectors for overexpression. Full-length cDNAs of *LIC*, *GSK1*, *SKETHA*, and *AtBIN2* without the stop codon were amplified by PCR from rice or *Arabidopsis* and cloned into pGADT7 or pGBDT7 vectors. All binary vector constructs were transformed into *Agrobacterium tumefaciens* strain GV3101 or EHA105, then transformed into rice calli by *A. tumefaciens*-mediated transfection [54], [55]. Primers are in Table S4.

For tobacco transformation, full-length cDNAs of *LIC* and *GSK1* were ligated into pBI121 and pRT105-3×flag vectors [56], respectively. The binary vector constructs were transformed into *A. tumefaciens* strain GV3101 and then transformed into tobacco by *A. tumefaciens*-mediated transfection.

### Protoplast Transient Expression Assay

Full-length *LIC* sequence was inserted into the pBI221 vector to generate pBI221-LIC. To generate the *BZR1pro:LUC* reporter gene, the *BZR1* promoter was amplified with the rice genomic DNA used as a template and then inserted into the pGEM-T Easy vector to produce pGEM-BZR1p. The *BZR1* promoter was released from pGEM-BZR1p by digestion with *Hin*dIII and *Bam*HI and inserted into the corresponding sites of the YY96 vector [57] to produce *BZR1pro:LUC*. The *ILI1pro:LUC* and *IBH1pro:LUC* reporter genes were constructed as for *BZR1pro:LUC*.

Isolation of *Arabidopsis* protoplasts and PEG-mediated transfection were as described [58]. The reporter constructs *BZR1pro:LUC*, *ILI1pro:LUC* and *IBH1pro:LUC*; effector plasmid; and *35S:GUS* construct (internal control) were co-transformed into protoplasts. After transformation, the protoplasts were incubated at 23°C for 12–15 h, then pelleted and resuspended in 100 µL of 1× CCLR buffer (Promega). For the ß-glucuronidase enzymatic assay, 5 µL extract was incubated with 50 µL 4-methylumbelliferyl ß-d-glucuronide assay buffer (50 mM sodium phosphate, pH 7.0, 1 mM ß-d-glucuronide, 10 mM EDTA, 10 mM ß-mercaptoethanol, 0.1% sarkosyl, 0.1% Triton X-100) at 37°C for 15 min, and the reaction was stopped by adding 945 µL of 0.2 M Na_2_CO_3_. For luciferase activity assay, 5 µL extract was mixed with 50 µL luciferase assay substrate (Promega), and activity was detected with use of a Modulus Luminometer/Fluometer with a luminescence kit (Promega). The reporter gene expression was expressed as relative ratio of LUC to ß-glucuronidase.

### Yeast Two-Hybrid Screening

The cDNA of *LIC* was cloned into the pGADT7 vector. The cDNAs of *GSK1*, *SKETHA*, and *AtBIN2* were cloned into pGBDT7 (Stratagene) and then transformed into yeast strain AH109. Transformants were screened for growth on medium lacking Leu, Trp, and His. Recovered clones were then assayed for LacZ activity by a filter lift assay. For the transactivation activity assay, *LIC*, *AtBIN2*, *GSK1*, and *SKETHA* were cloned into the pGBDT7 vector and co-transformed with pGADT7 into yeast cells. Yeast that could grow on SD/-Leu/-Trp/-His medium with ß-galactosidase activity exhibited transactivation activity.

Western blot analysis involved extracts prepared from yeast cells as described [14]. The yeast cells were collected, ground to a fine powder in liquid nitrogen, and further ground in cold grinding buffer (50 mM HEPES (pH 7.4), 10 mM EDTA, 0.1% Triton X-100, 1 mM PMSF). After the addition of an equal volume of 2× sample buffer, the samples were boiled for 10 min, separated by 15% SDS–PAGE, and transferred to a polyvinylidene fluoride membrane. The blots were incubated with the antibodies mouse anti-Myc (Neo-Marker, UK) or mouse anti-HA (Santa Cruz, Germany), then goat anti-mouse IgG HRP-conjugated secondary antibody (Santa Cruz, Germany).

### ChIP and EMSA

Chromatin immunprecitipation (ChIP) was performed as described [26] with 3-week-old seedlings. The antibody polyclonal anti-BZR1 or anti-LIC was used for immunoprecipitation. Untagged purified LIC protein was used to inject rabbit, and polyclonal serum was affinity-purified with its target antigen. ChIP products were analyzed by quantitative real-time PCR, and enrichment was calculated as the ratio of transgenic to wild-type sample or BR-treated and control seedlings. Data are mean±SD from 3 biological replicates. The primers for UBQ5 (LOC_Os04g57220) promoter were 5′-TATCCAACATGAATGCCACA-3′ and 5′-CAGCACGAGATGAGTAAAACAA-3′. Sequences used in bioinformatics analysis are in Table S3.

EMSA was performed essentially as described [59]. Briefly, the OsBZR1 coding region was cloned into a maltose-binding protein (MBP) fusion vector (pETMALc-H vector, Pryor and Leiting, 1997) with the primers for OsBES1NAsp718, 5′-CTCGGTACCGGAGCTGGTGGGTATGACGTC-3′, and OsBES1CHind3, 5′-CGCAAGCTTTCATTTCGCGCCGACGCCGAGC-3′. The recombinant MBP–OsBZR1 was purified from *Escherichia coli* with amylose resin (NEB, http://www.neb.com) according to the manufacturer's instructions [60]. The coding sequence of *LIC* was cloned into the expression vector pGEX-4T-1 [10]. The construct was transformed into *E. coli* BL21 (DE3). Cells were grown at 30°C and induced by the addition of isopropyl β-d-thiogalactopyranoside at a final concentration of 1 mM when the optical density (OD)_600_ of the cultured cells was 0.5–0.9. The fusion protein was purified with Glutathione Sepharose 4B (GE Healthcare). The nucleotide sequences of the double-stranded oligonucleotides for EMSA were for LIC P1 (5′-CGA CGT CGT GCG GCC GCG-3′ and 5′-CGC GGC CGC ACG ACG TCG-3′) and LIC P2 (5′-CGG GCG CGT GTG TGG CGG-3′ and 5′-CCG CCA CAC ACG CGC CCG-3′). The oligonucleotides were annealed and then labeled with the Biotin 3′ End DNA Labeling Kit (Pierce). Standard reaction mixtures (20 µL) for EMSA contained 2 µg purified proteins, 2 µL biotin-labeled annealed oligonucleotides, 2 µL 10×binding buffer (100 mM Tris, 500 mM KCl, 10 mM DTT, pH 7.5), 1 µL 50% glycerol, 1 µL 1% NP-40, 1 µL 1 M KCl, 1 µL 100 mM MgCl_2_, 1 µL 200 mM EDTA, 1 µL 1 µg/µL poly (dI-dC) and 8 µL ultrapure water. The reactions were incubated at room temperature (25°C) for 20 min and loaded onto a 10% native polyacrylamide gel containing 45 mM Tris, 45 mM boric acid, 1 mM EDTA, pH 8.3. The gel was sandwiched and transferred to an N^+^ nylon membrane (Millipore) in 0.5× TBE buffer at 380 mA in a 4°C refrigerator for 60 min. The detection of biotin-labeled DNA by chemiluminescence followed the manual of the LightShift Chemiluminescent EMSA Kit (PIERCE).

### Confocal Microscopy and Quantification of Protein Fluorescent Signal

GFP fluorescence was visualized under a confocal microscope (Zeiss LSM510 META, Germany) equipped with an argon laser (488 nm). GFP was excited by an Argon laser at 488 nm, and images were acquired using a 512b Roper Cascade EMCCD camera and MetaMorph software (Molecular Devices, Sunnyvale, CA). Images of LIC-, LICm-, and LICp-GFP were obtained with identical image acquisition settings. A series of images at different points along the z-axis were collected from the top to the bottom. Projection of the z-series of images results in a 3D view of the cell. To quantify the effect of 24-eBL on OsLIC-GFP localization in the time-course experiment, images were obtained with a 500-ms exposure time. Quantification of the fluorescent protein signal involved use of ImageJ (http://rsb.info.nih.gov/ij). To measure the ratio of nuclear to cytoplasmic signals (N/C ratio) for LIC-GFP for each cell, small areas were drawn, and measurements of integrated densities were taken from representative areas within the nucleus, cytoplasm, and background (central vacuole) of each cell. Each sample of at least 20 cells was measured 3 times; the average N/C ratio were then calculated [42].

### Western Blot Analysis and Kinase Assay *In Vitro*

Total protein samples were extracted from 2-week-old rice seedlings with 2× SDS loading buffer; cytoplasmic and nuclear fractions were extracted as described [20], [61]. Tissues were lysed with use of a buffer (20 mM Tris-HCl, pH 7.0, 250 mM sucrose, 25% glycerol, 20 mM KCl, 2 mM EDTA, 2.5 mM MgCl_2_, 30 mM ß-mercaptoethanol, 13-protease inhibitor cocktail, and 0.7% Triton X-100) and fractionated by centrifugation at 3000×*g*. The supernatant was taken as the cytosolic fraction. The pellet was further washed with a resuspension buffer (20 mM Tris-HCl, pH 7.0, 25% glycerol, 2.5 mM MgCl_2_, and 30 mM ß-mercaptoethanol) and reconstituted as the nuclear fraction. All proteins were separated on SDS-PAGE gels, transferred to a nitrocellulose membrane, and probed with anti-LIC antibody. For the *in vitro* kinase assay, purified LIC-GST protein was incubated with BIN2/GSK1 protein or λ-phosphorylase 1 at 30°C for 30 min and loaded onto SDS-PAGE gels. SDS-PAGE gels of 8% or 15% were used to analyze the total LIC level or its phosphorylation, respectively. The proteins were transferred to a nitrocellulose membrane until the 35-kDa protein marker ran out of the gel during electrophoresis.

### RNA Hybridization *In Situ*

Tissues were fixed in 4% (w/v) paraformaldehyde and 0.25% glutaraldehyde in 0.1 M sodium phosphate buffer; samples were vacuum-infiltrated for 30 min and then stored overnight at 4°C. The dehydrated samples after a graded ethanol series were embedded in Paraplast Plus (Oxford Labware, St. Louis, MO). A fragment of 232 bp was amplified from the second exon of *LIC* with the primers 5′-GGATCCGCAAGTACGGAGCGCAGTG-3′ and 5′-AAGCTTTTCG CAGGACCAGGAGCA-3′, subcloned into the pGEM-T-easy vector (Promega), and used as a template for RNA probe synthesis. A fragment of *BZR1* was amplified with the primers 5′-ATCAGGAAGCCGGACTGGG-3′ and 5′-GGTTGACGAGGTTGTAGGTGGG-3′. Hybridization *in situ* with digoxigenin-labeled sense or antisense RNA of *LIC* and *BZR1* was conducted as described [62].

### Bioinformatics Analysis of the Putative Binding Motif

MEME software (http://meme.sdsc.edu/meme/cgi-bin/meme.cgi) was used to find recurrent motifs among multiple sequences in Affymetrix microarray data for the *LIC* antisense lines that were up- or downregulated by at least 2-fold [10]. We extracted 1-kb genomic sequences upstream of 1,175 genes to screen the potential motifs. Differentially expressed genes were divided into those up- or downregulated. Randomly generated sequences of the same length were used as controls to remove false-positive results.

### Accession Numbers

Sequence data from this article can be found in the GenBank or EMBL database under the following accession numbers: LIC, Os06g49080; GSK1, Os01g10840; SKETHA, Os06g35530; ILI1, Os04g54900; Os IBH1, Os04g0660100.

## Supporting Information

Figure S1 Identification of *lic-1* mutant and *LIC*-overexpressing lines. (A) A diagram of the T-DNA insertion site in the *lic-1* mutant and the primers used in the identification of the mutant. LB represents the left border primer in T-DNA, LP and RP represent the left and right primers for *LIC* respectively. P1+P2 represent primers used to amplify the N-terminal fragment of *LIC* and P1+P3 represent primers used to amplify full-length *LIC*. (B) PCR of genomic DNA to amplify T-DNA with primers LB+RP and *LIC* with primers LP+RP. Italicized numbers 9, 17 and 22 indicate homozygous mutants. (C) PCR of cDNA to amplify full-length *LIC* and the N-terminal fragment of *LIC* in the *lic-1* mutant. (D) Quantitative RT-PCR analysis of *LIC* RNA levels in antisense lines and overexpressing lines. Data are mean ± SD (*n* = 3).
(TIF)

Figure S2 Comparative morphology of the *lic-1* mutant and the transgenic lines. (A) Gross morphologic features of LIC overexpressors and the *lic-1* mutant (40 days old). *LIC*-overexpressing lines (OX1) and the *lic-1* mutant showed dwarfism and erect leaves. The antisense line 2 (AS2) and BR-deficient mutant *d2* are controls. Bar = 20 cm. (B) Quantification of leaf angles in the wild type, *lic-1* mutant and OX1; AS2 and *d2* are controls. Data are mean±SE of 50 measured plants.
(TIF)

Figure S3 Phenotypes of *lic-1* mutant and *LIC* transgenic lines. *lic-1* mutant and *LIC*-overexpressing line 2 (OX2) show erect leaves and antisense line 3 (AS3) an increased leaf angle.
(TIF)

Figure S4 BR marker genes expression in transgenic lines. Quantitative RT-PCR analysis of the mRNA level of BR synthetic genes *D2*, *D11*, *BRD1* and the receptor gene *BRI1* in the wild type, *LIC* antisense line 2 (AS2) and *lic-1* mutant. Data are mean ± SD (*n* = 3). *P<0.05 and **P<0.01 compared with the wild type as determined by Student's *t* test.
(TIF)

Figure S5 Rice root growth at different concentrations of BR. (A) BR sensitivity of the *lic-1* mutant and the *LIC*-overexpressing lines in root growth. The upper panel represents treatment without BR, and the bottom panel represents 1 µM BR treatment; OX1, *LIC*-overexpressing line 1; AS2, *LIC* antisense line 2. Bar = 1 cm. (B) Quantification of primary root length under different concentrations of BR. Data are mean ±SD of root length in 30 plants. (C) BR promoted root growth at low levels (<1 nM) and restrained root elongation at high levels (>100 nM). Bar = 2 cm.
(TIF)

Figure S6 Identification of hybrid generations of a *LIC* antisense line and a *BZR1* RNAi line. (A) Identification of the *BZR1* RNAi vector and the *LIC* antisense vector in hybrid generations. H1, H2 and H3 represent hybrid generations and CK indicates the *BZR1* RNAi line or the *LIC* antisense line as a positive control. (B) Quantitative RT-PCR analysis of *LIC* and *BZR1* RNA levels in parent lines and hybrid generations. Data are mean ± SD (*n* = 3).
(TIF)

Figure S7 Western blot analysis of protein expression in the yeast cells. (A) LIC interacted with AtBIN2 and rice orthologs in yeast cells. Left panel, LIC interacted with AtBIN2, OsGSK1 and OsSKETHA in a yeast two-hybrid assay; pGADT7-DWF1– and pGBDT7-GSR1–co-transformed yeast served as a positive control [63] and AD– and BD vector–co-transformed yeast as a negative control. Middle panel, mutated LIC failed to interact with BIN2/GSK1/SKETHA, pGADT7-LICm- and pGBDT7-co-transformed yeast served as a negative control. Right panel, yeast cells transformed with a single protein served as a negative control. (B) Western blot analysis with an anti-HA tag antibody. Protein was extracted from yeast co-transformed with LIC/LICm/LICp and GSK1 or yeast co-transformed with the AD and BD vectors. (C) Immunoblotting analysis with an anti-Myc tag antibody. Protein was extracted from yeast co-transformed with BIN2/GSK1/SKETHA and LICm or yeast co-transformed the AD and BD vectors.
(TIF)

Figure S8 EMSA to test LIC binding to the predicted motifs. (A) Putative DNA motifs to which LIC binds (denoted as S1–14) as predicted by use of microarray chip gene promoters and MEME software (see Materials and Methods). (B) EMSA to illustrate LIC binding to S1–3. Lane 1 shows the band shift caused by S1, lane 2 the band shift caused by S2, lane 3 the band shift caused by S3, and lanes 4–6 unlabeled S1–3 (denoted as Co1-3), which served as competitive probes that weakened the intensity of the shifted bands of S1–3. Lanes 7–9, mutated S1–3, denoted as MS1 (GAAAATG), MS2 (TCGAAAA ,) and MS3 (CTAAAAT) respectively, eliminated the shifted bands. (C) Putative DNA motifs to which LIC binds as predicted from ChIP sequences. Letter probability of every site is shown on the right. (D) LIC bound to the sequence CTCGC marked as S. M1 ( ATCGCG), M2 (CTCGCT ) and M3 (CAAAAG) were the mutated probes. Co represented the competitive unlabeled S sequence.
(TIF)

Figure S9 Specificity of the anti-LIC antibody and the anti-BZR1 antibody used in the ChIP assay. Left, western blot analysis with the LIC antibody displayed one specific band for the total protein fraction; LIC protein was decreased in antisense lines and increased in overexpressing lines. Right, western blot with the BZR1 antibody displayed one specific band for wild-type proteins.
(TIF)

Figure S10 Expression patterns of *LIC* and *BZR1*. (A) Expression patterns of *LIC* and *BZR1* in various organs in rice (S, shoot; R, root; ST, stem; P, panicle; L, leaf; LS, leaf sheath). Data are mean ± SD (*n* = 3). (B) *LIC* and *BZR1* expression patterns during seed development and in leaves (Data analyzed by use of electronic fluorescent pictographic software, http://www.bar.utoronto.ca/efp/cgi-bin/efpWeb.cgi). The color scale illustrates the microarray signal level. YL, young leaf; ML, mature leaf.
(TIF)

Figure S11 Phylogenic tree of rice LIC (Os06g49080) and related proteins in other model species. The sequence of *LIC* was used in BLAST searches of NCBI databases (http://130.14.29.110/blast/, nr, est, httg, gss, and wgs databases, default values). Midpoint-rooted neighbor-joining trees were constructed with full-length protein sequences by use of MEGA 3.1 (http://www.megasoftware.net/index.html) [64]. The variables were poisson correction, pairwise deletion and bootstrap (1000 replicates; random seed). Blue box: genes of dicots; red box: genes of monocots.
(TIF)

Table S1 Number of seeds per panicle and leaf angle for the progenies of antisense line 2 and *lic-1* hybrid lines.
(DOC)

Table S2 Phenotypes of *LIC* transgenic rice lines and gain-of-function mutants.
(DOC)

Table S3 Sequences from the ChIP assay for motif searches.
(DOC)

Table S4 Primers used in this study.
(DOC)
