# Supplementary figures and images for: Dynamics of Brassinosteroid Response Modulated by Negative Regulator LIC in Rice
Source: PLoS Genet. 2012 Apr 26;8(4):e1002686. doi: 10.1371/journal.pgen.1002686 (PMC3343102; doi:10.1371/journal.pgen.1002686)

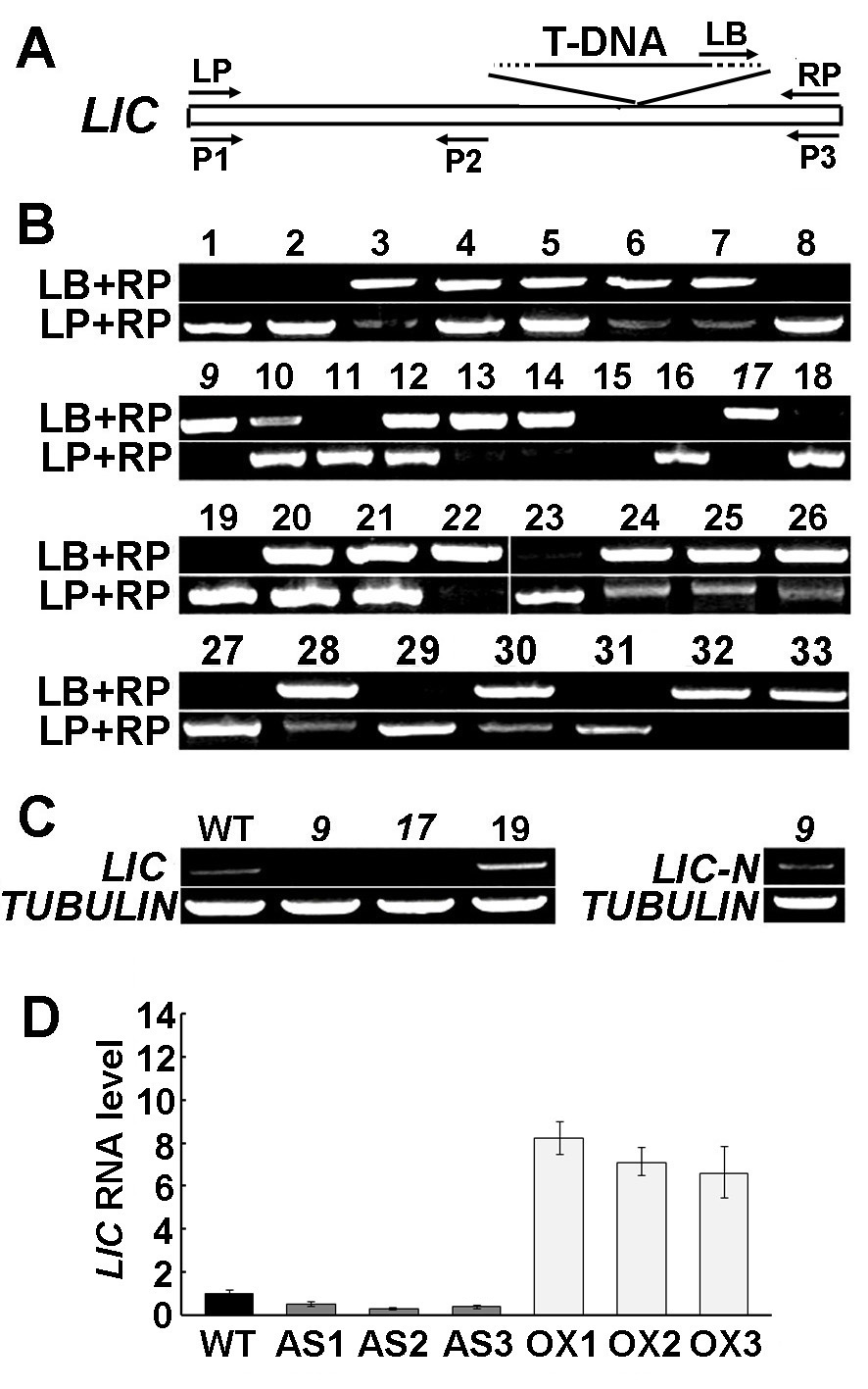

Supplement: Figure S1 — Identification of lic-1 mutant and LIC-overexpressing lines. (A) A diagram of the T-DNA insertion site in the lic-1 mutant and the primers used in the identification of the mutant. LB represents the left border primer in T-DNA, LP and RP represent the left and right primers for LIC respectively. P1+P2 represent primers used to amplify the N-terminal fragment of LIC and P1+P3 represent primers used to amplify full-length LIC. (B) PCR of genomic DNA to amplify T-DNA with primers LB+RP and LIC with primers LP+RP. Italicized numbers 9, 17 and 22 indicate homozygous mutants. (C) PCR of cDNA to amplify full-length LIC and the N-terminal fragment of LIC in the lic-1 mutant. (D) Quantitative RT-PCR analysis of LIC RNA levels in antisense lines and overexpressing lines. Data are mean ± SD (n = 3). (TIF) [file pgen.1002686.s001.tif]

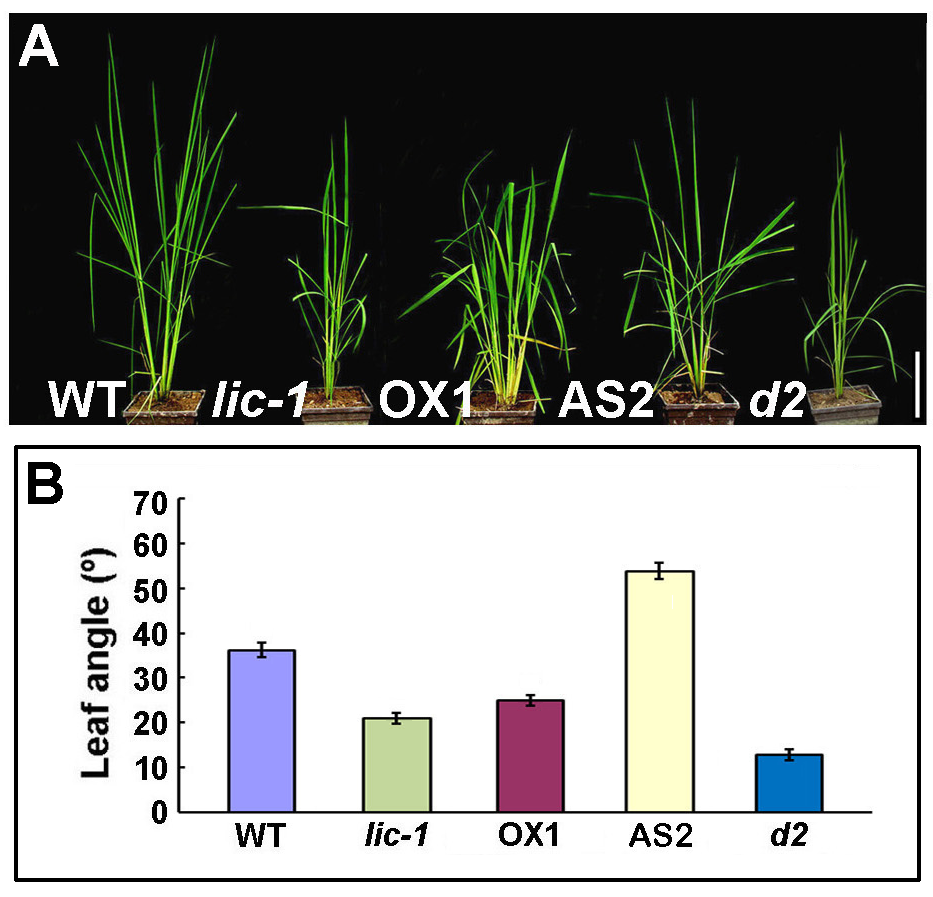

Supplement: Figure S2 — Comparative morphology of the lic-1 mutant and the transgenic lines. (A) Gross morphologic features of LIC overexpressors and the lic-1 mutant (40 days old). LIC-overexpressing lines (OX1) and the lic-1 mutant showed dwarfism and erect leaves. The antisense line 2 (AS2) and BR-deficient mutant d2 are controls. Bar = 20 cm. (B) Quantification of leaf angles in the wild type, lic-1 mutant and OX1; AS2 and d2 are controls. Data are mean±SE of 50 measured plants. (TIF) [file pgen.1002686.s002.tif]

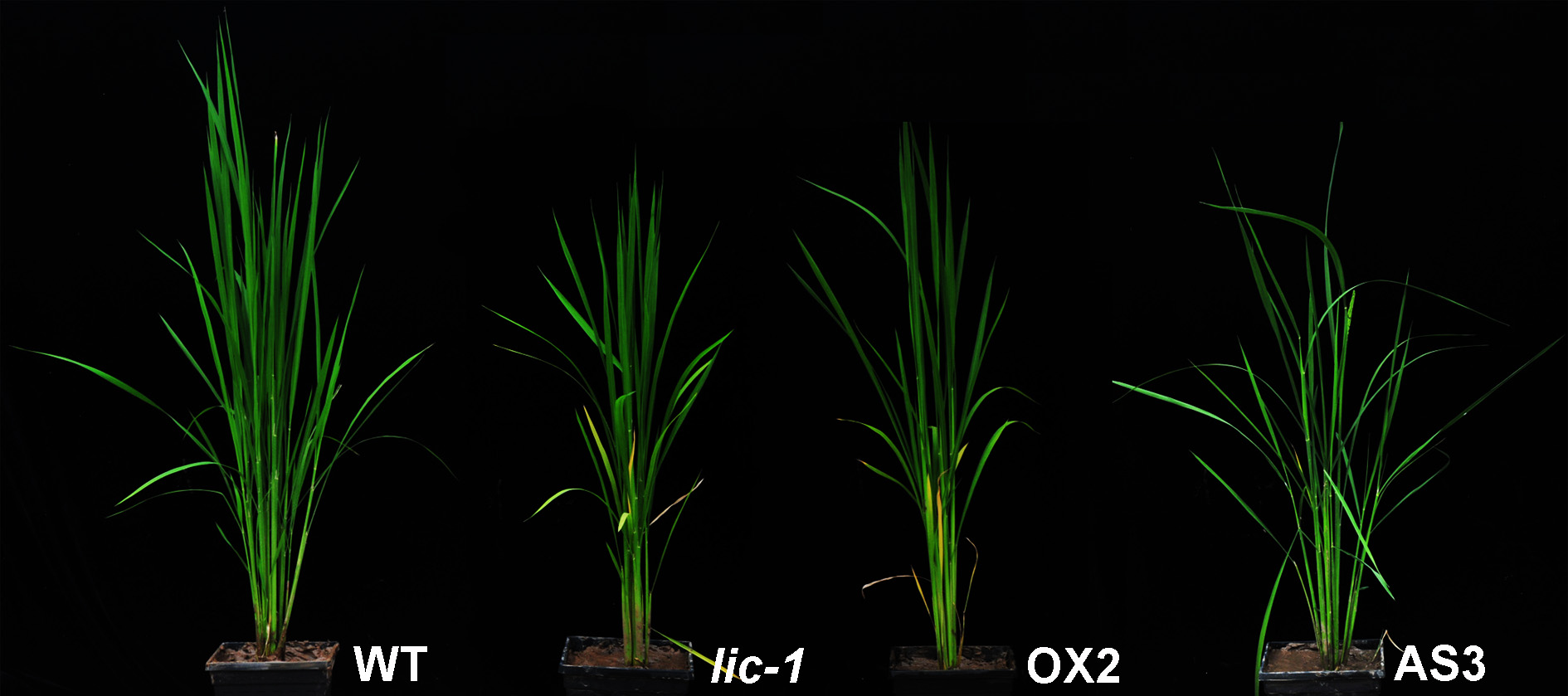

Supplement: Figure S3 — Phenotypes of lic-1 mutant and LIC transgenic lines. lic-1 mutant and LIC-overexpressing line 2 (OX2) show erect leaves and antisense line 3 (AS3) an increased leaf angle. (TIF) [file pgen.1002686.s003.tif]

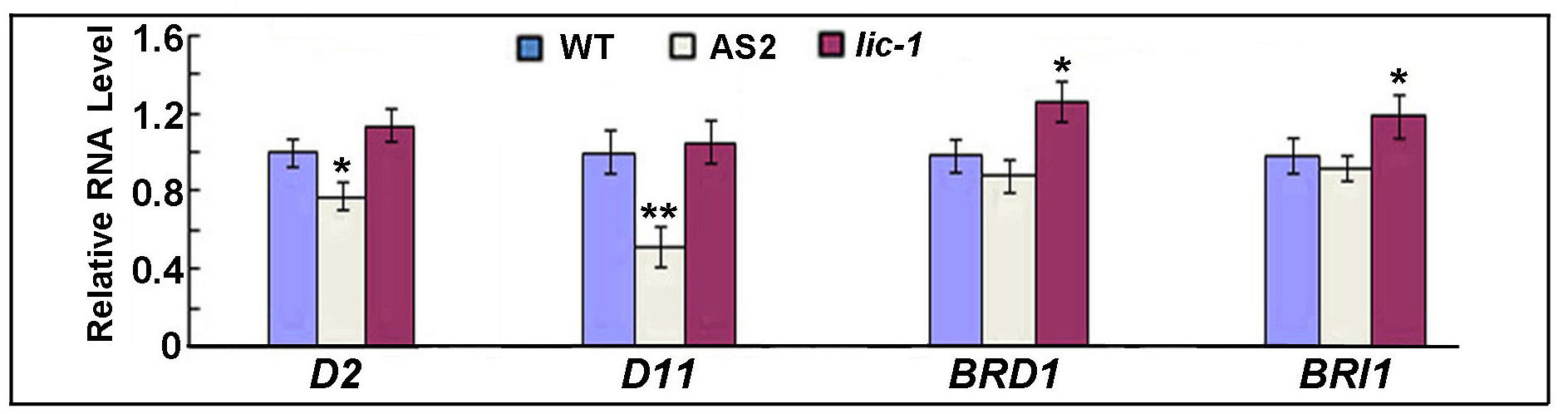

Supplement: Figure S4 — BR marker genes expression in transgenic lines. Quantitative RT-PCR analysis of the mRNA level of BR synthetic genes D2, D11, BRD1 and the receptor gene BRI1 in the wild type, LIC antisense line 2 (AS2) and lic-1 mutant. Data are mean ± SD (n = 3). *P<0.05 and **P<0.01 compared with the wild type as determined by Student's t test. (TIF) [file pgen.1002686.s004.tif]

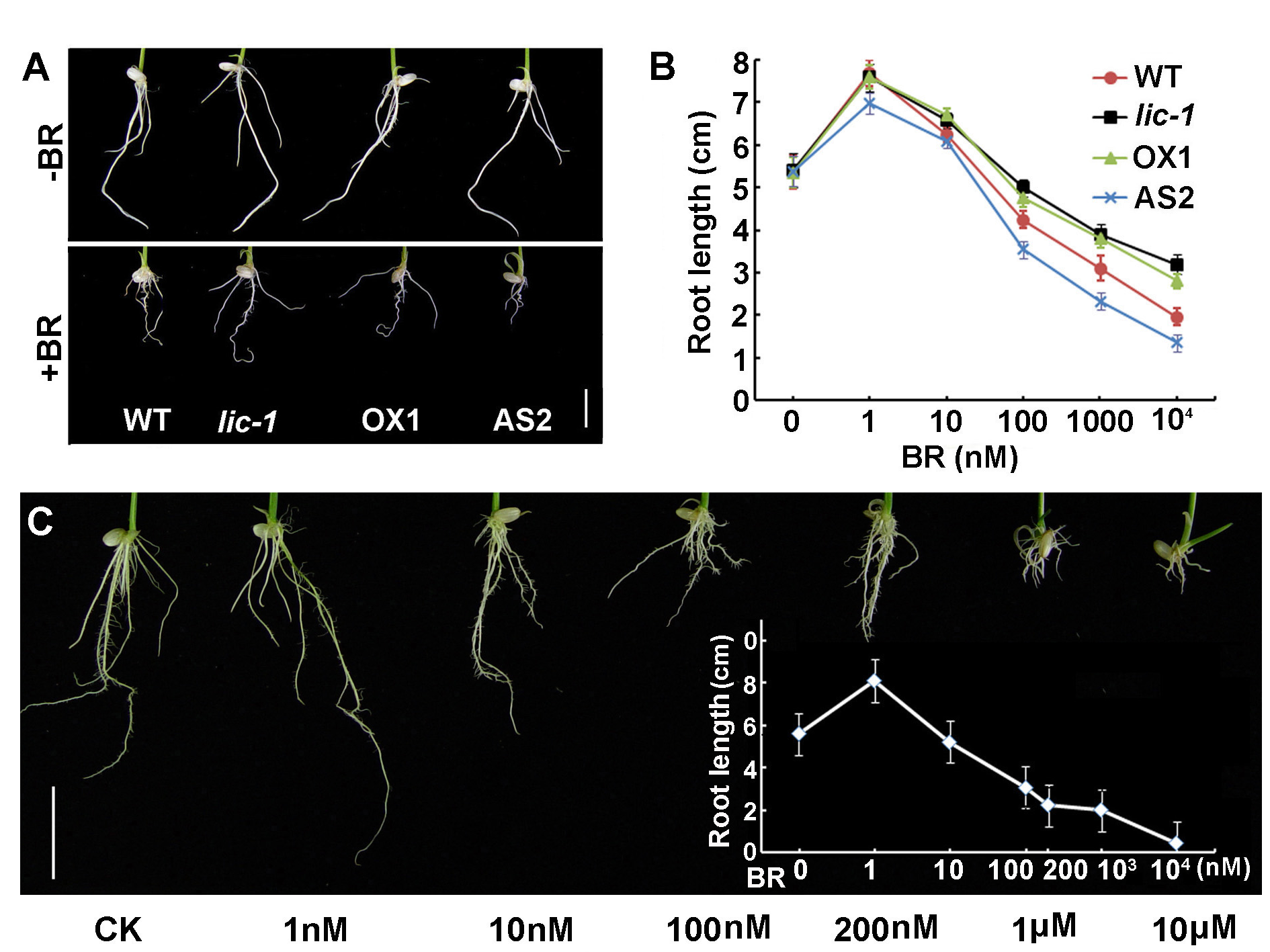

Supplement: Figure S5 — Rice root growth at different concentrations of BR. (A) BR sensitivity of the lic-1 mutant and the LIC-overexpressing lines in root growth. The upper panel represents treatment without BR, and the bottom panel represents 1 µM BR treatment; OX1, LIC-overexpressing line 1; AS2, LIC antisense line 2. Bar = 1 cm. (B) Quantification of primary root length under different concentrations of BR. Data are mean ±SD of root length in 30 plants. (C) BR promoted root growth at low levels (<1 nM) and restrained root elongation at high levels (>100 nM). Bar = 2 cm. (TIF) [file pgen.1002686.s005.tif]

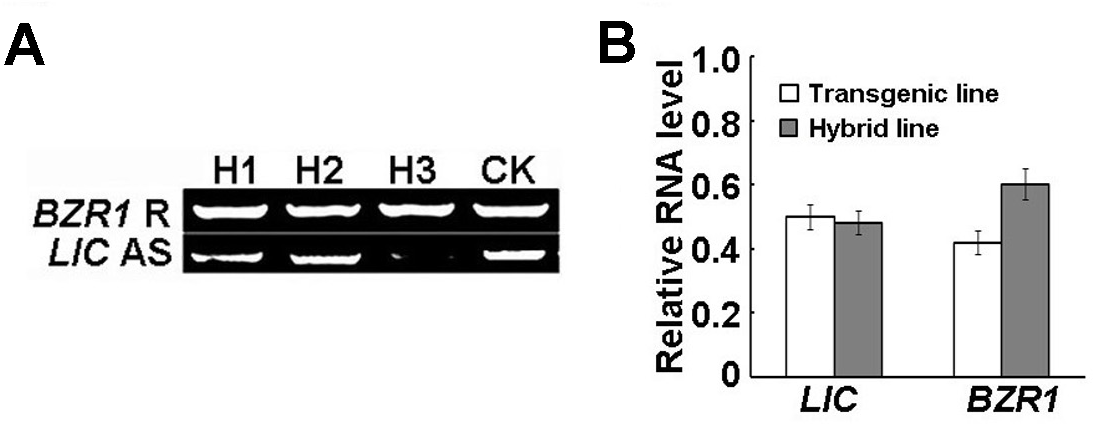

Supplement: Figure S6 — Identification of hybrid generations of a LIC antisense line and a BZR1 RNAi line. (A) Identification of the BZR1 RNAi vector and the LIC antisense vector in hybrid generations. H1, H2 and H3 represent hybrid generations and CK indicates the BZR1 RNAi line or the LIC antisense line as a positive control. (B) Quantitative RT-PCR analysis of LIC and BZR1 RNA levels in parent lines and hybrid generations. Data are mean ± SD (n = 3). (TIF) [file pgen.1002686.s006.tif]

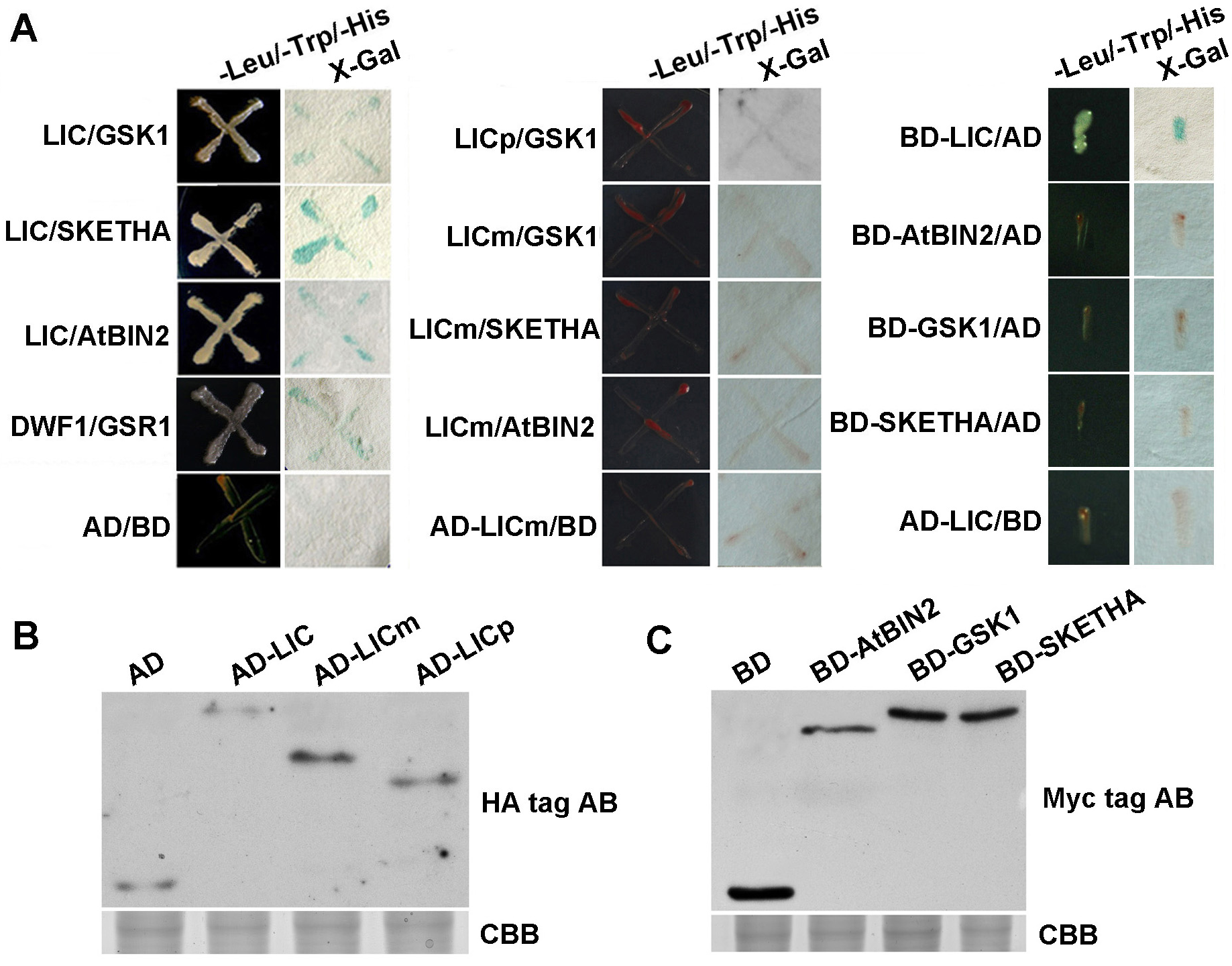

Supplement: Figure S7 — Western blot analysis of protein expression in the yeast cells. (A) LIC interacted with AtBIN2 and rice orthologs in yeast cells. Left panel, LIC interacted with AtBIN2, OsGSK1 and OsSKETHA in a yeast two-hybrid assay; pGADT7-DWF1– and pGBDT7-GSR1–co-transformed yeast served as a positive control [63] and AD– and BD vector–co-transformed yeast as a negative control. Middle panel, mutated LIC failed to interact with BIN2/GSK1/SKETHA, pGADT7-LICm- and pGBDT7-co-transformed yeast served as a negative control. Right panel, yeast cells transformed with a single protein served as a negative control. (B) Western blot analysis with an anti-HA tag antibody. Protein was extracted from yeast co-transformed with LIC/LICm/LICp and GSK1 or yeast co-transformed with the AD and BD vectors. (C) Immunoblotting analysis with an anti-Myc tag antibody. Protein was extracted from yeast co-transformed with BIN2/GSK1/SKETHA and LICm or yeast co-transformed the AD and BD vectors. (TIF) [file pgen.1002686.s007.tif]

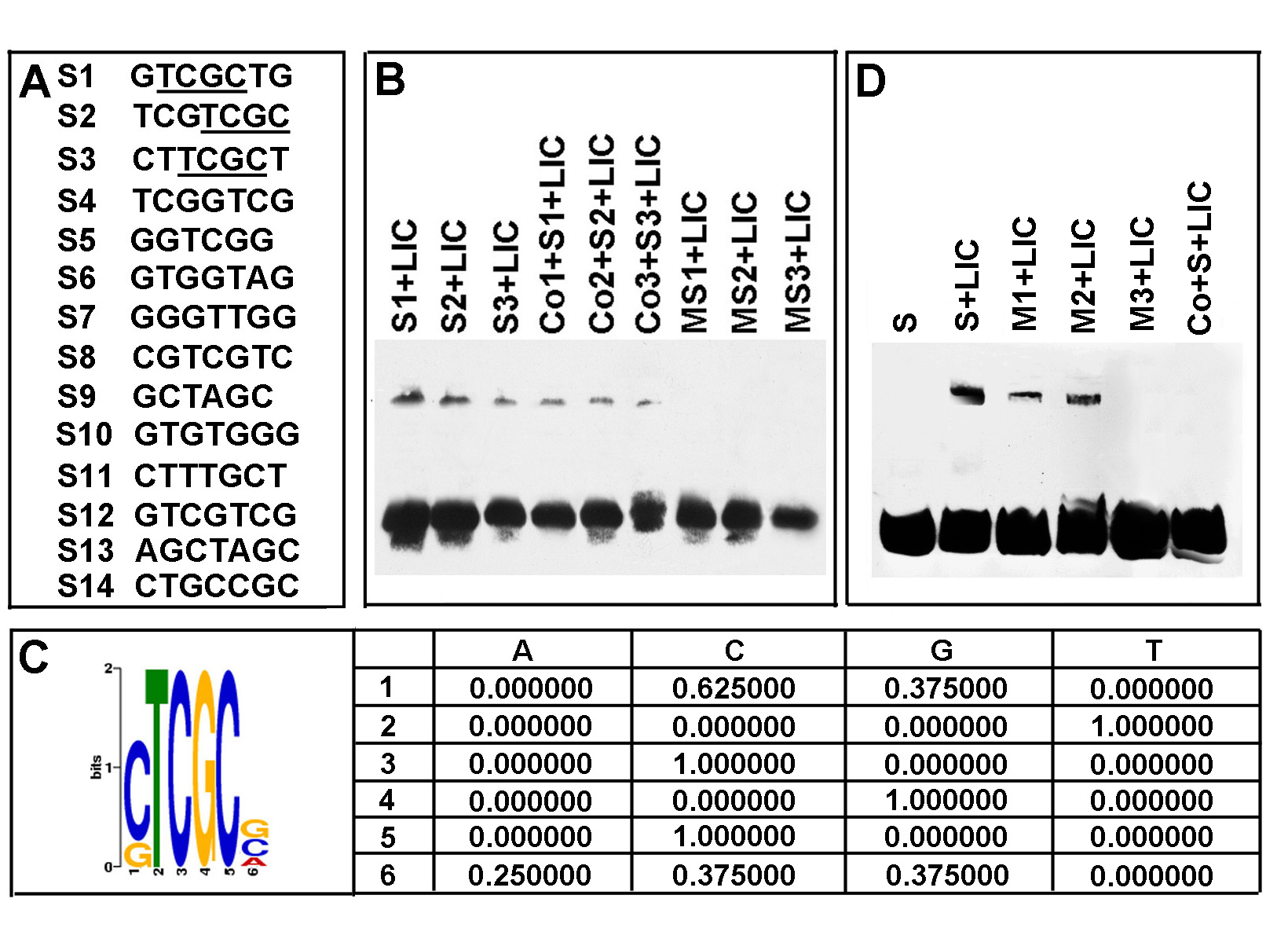

Supplement: Figure S8 — EMSA to test LIC binding to the predicted motifs. (A) Putative DNA motifs to which LIC binds (denoted as S1–14) as predicted by use of microarray chip gene promoters and MEME software (see Materials and Methods). (B) EMSA to illustrate LIC binding to S1–3. Lane 1 shows the band shift caused by S1, lane 2 the band shift caused by S2, lane 3 the band shift caused by S3, and lanes 4–6 unlabeled S1–3 (denoted as Co1-3), which served as competitive probes that weakened the intensity of the shifted bands of S1–3. Lanes 7–9, mutated S1–3, denoted as MS1 (GAAAATG), MS2 (TCGAAAA ,) and MS3 (CTAAAAT) respectively, eliminated the shifted bands. (C) Putative DNA motifs to which LIC binds as predicted from ChIP sequences. Letter probability of every site is shown on the right. (D) LIC bound to the sequence CTCGC marked as S. M1 ( ATCGCG), M2 (CTCGCT ) and M3 (CAAAAG) were the mutated probes. Co represented the competitive unlabeled S sequence. (TIF) [file pgen.1002686.s008.tif]

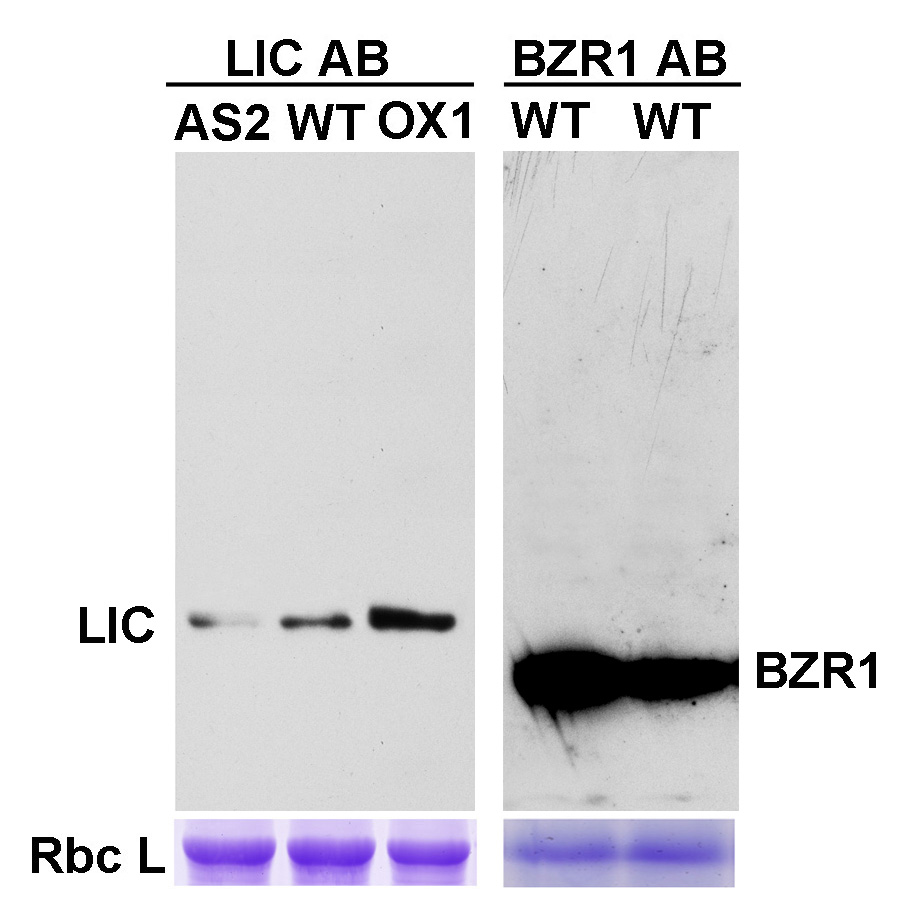

Supplement: Figure S9 — Specificity of the anti-LIC antibody and the anti-BZR1 antibody used in the ChIP assay. Left, western blot analysis with the LIC antibody displayed one specific band for the total protein fraction; LIC protein was decreased in antisense lines and increased in overexpressing lines. Right, western blot with the BZR1 antibody displayed one specific band for wild-type proteins. (TIF) [file pgen.1002686.s009.tif]

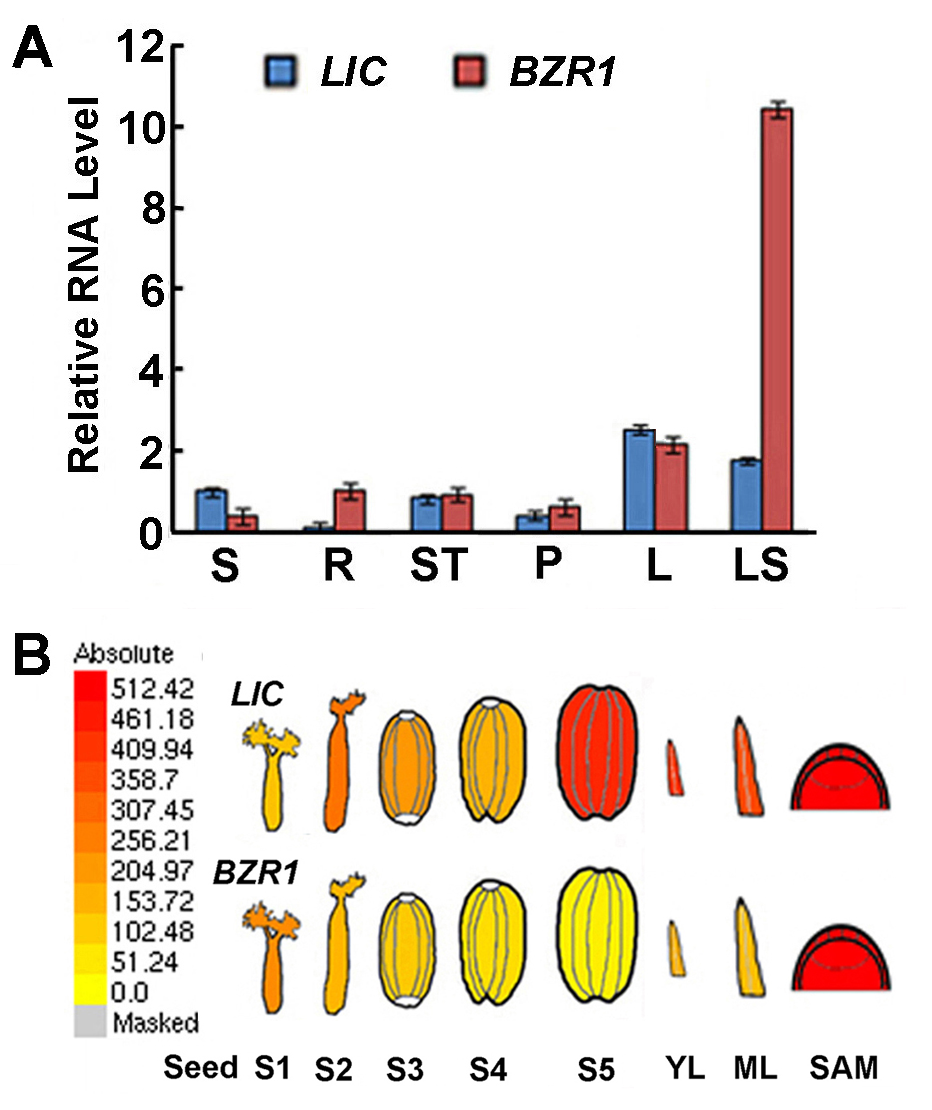

Supplement: Figure S10 — Expression patterns of LIC and BZR1. (A) Expression patterns of LIC and BZR1 in various organs in rice (S, shoot; R, root; ST, stem; P, panicle; L, leaf; LS, leaf sheath). Data are mean ± SD (n = 3). (B) LIC and BZR1 expression patterns during seed development and in leaves (Data analyzed by use of electronic fluorescent pictographic software, http://www.bar.utoronto.ca/efp/cgi-bin/efpWeb.cgi). The color scale illustrates the microarray signal level. YL, young leaf; ML, mature leaf. (TIF) [file pgen.1002686.s010.tif]

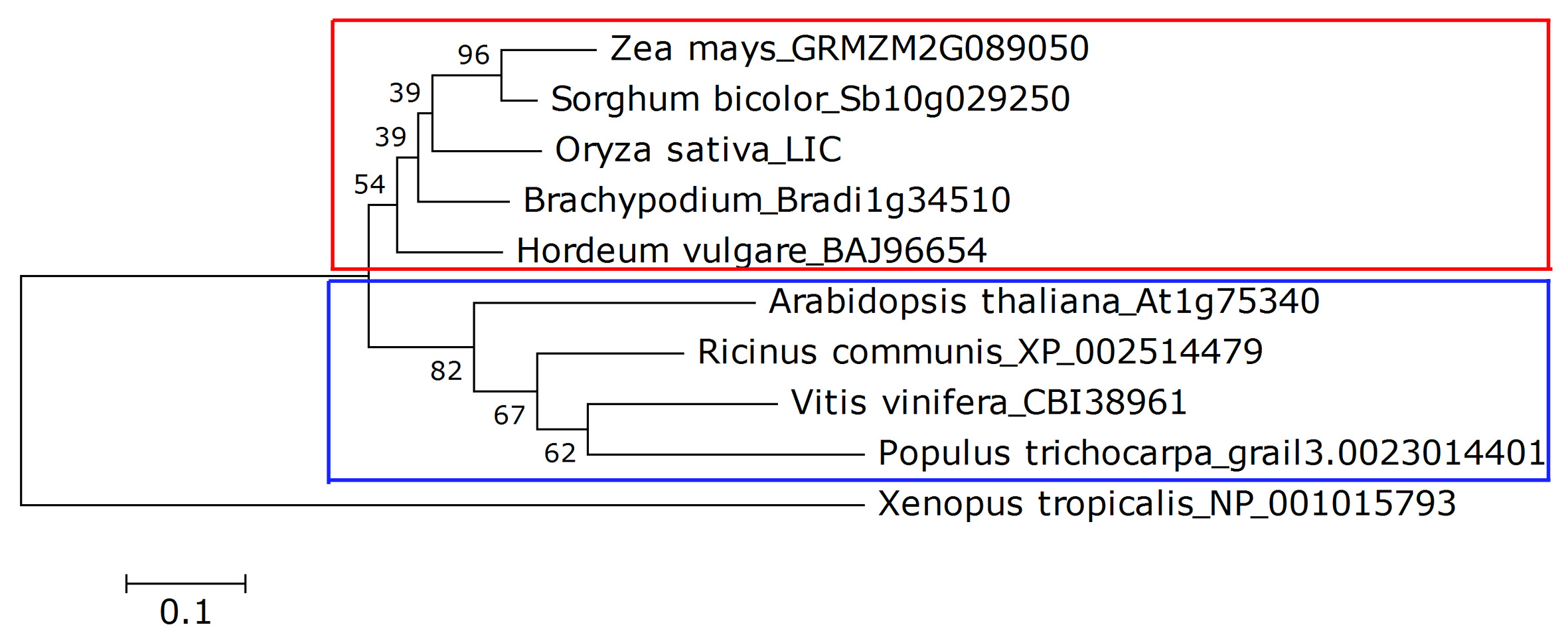

Supplement: Figure S11 — Phylogenic tree of rice LIC (Os06g49080) and related proteins in other model species. The sequence of LIC was used in BLAST searches of NCBI databases (http://130.14.29.110/blast/, nr, est, httg, gss, and wgs databases, default values). Midpoint-rooted neighbor-joining trees were constructed with full-length protein sequences by use of MEGA 3.1 (http://www.megasoftware.net/index.html) [64]. The variables were poisson correction, pairwise deletion and bootstrap (1000 replicates; random seed). Blue box: genes of dicots; red box: genes of monocots. (TIF) [file pgen.1002686.s011.tif]
