# Supplementary material for: Dynamics of Brassinosteroid Response Modulated by Negative Regulator LIC in Rice
Source: PLoS Genet. 2012 Apr 26;8(4):e1002686. doi: 10.1371/journal.pgen.1002686 (PMC3343102; doi:10.1371/journal.pgen.1002686)
Supplement: Table S1 — Number of seeds per panicle and leaf angle for the progenies of antisense line 2 and lic-1 hybrid lines. (DOC) [file pgen.1002686.s012.doc]

**Supplemental Table S1. Number of seeds per panicle and leaf angle for the progenies of antisense line 2 and *lic-1* hybrid lines.**

|  | WT | AS2 | *lic-1* | AS2x*lic-1* |
| --- | --- | --- | --- | --- |
| Number of plants  mean+SD | 6 | 6 | 6 | 6 |
| Number of seeds/panicle  mean+SD | 118.2+12.3 | 68.3+12.1 | 115.9+13.2 | 75.1+16.1 |
| Leaf angle (°)  mean+SD | 35.6+7.2 | 58.8+8.5 | 20.0+5.4 | 48.3+6.5 |
