# Supplementary material for: Dynamics of Brassinosteroid Response Modulated by Negative Regulator LIC in Rice
Source: PLoS Genet. 2012 Apr 26;8(4):e1002686. doi: 10.1371/journal.pgen.1002686 (PMC3343102; doi:10.1371/journal.pgen.1002686)
Supplement: Table S2 — Phenotypes of LIC transgenic rice lines and gain-of-function mutants. (DOC) [file pgen.1002686.s013.doc]

**Supplemental Table S2. Phenotypes of *LIC* transgenic rice lines and gain-of-function mutants.**

|  | WT | AS1 | AS2 | AS3 | OX1 | OX2 | OX3 | *lic-1* |
| --- | --- | --- | --- | --- | --- | --- | --- | --- |
| Number of plants | 15 | 15 | 15 | 15 | 15 | 15 | 15 | 15 |
| Height(cm) mean+SD | 120+15 | 68+8 | 70+11 | 71+12 | 71+11 | 72+13 | 86+15 | 62+9 |
| Leaf angle(°) mean+SD | 35+5 | 46+5 | 65+9 | 60+11 | 20+6 | 22+5 | 25+4 | 19+4 |
