# Supplementary material for: Dynamics of Brassinosteroid Response Modulated by Negative Regulator LIC in Rice
Source: PLoS Genet. 2012 Apr 26;8(4):e1002686. doi: 10.1371/journal.pgen.1002686 (PMC3343102; doi:10.1371/journal.pgen.1002686)
Supplement: Table S3 — Sequences from the ChIP assay for motif searches. (DOC) [file pgen.1002686.s014.doc]

**Supplemental Table S3. Sequences from the ChIP assay for motif searches.**

| **1** | atcacgattgagcaggtagctcgatctgctacataacgtactcagaaattaattaatttatatttttaagaaacacggtataaacacaaacgtt**ctcgc**gtgtgacactcacctatatgagcatttttagaagactgggcatattgacgtttcgttgtcgacgggtacgtcgtccactac |
| --- | --- |
| **2** | tttttaataaaaaagaataatttggattgtttttgtctctcgttgtcaactaggtattataagacaccggatcttgctcaggacatttgtgtcagagccgtgttc**ctcgc**tctcttccctttcctttttttctctctctaaaatcgcaacccacaaatccttctgatccgtttgtgtcag |
| **3** | tcatcaatcatgttccatgcctgggccgccgtcctagcgccggccacttgttgcagcacctcccttgatagagaggagaagatgaatccgagcacttgctggtcctgaacaaaccatgtc |
| **4** | gaaccgaact**ctcgc**atgcatatgcatgcaaatgcaggcacggacaagcggcatgattggtgatgatcatgtacgtattcgttgtgtcgttctaatgtgtatagttccaaaacctgatcgtcttaaccaacatgatt |
| **5** | ctcctcagtcgtc**ctcgc**accgcaacacatgctcaagactattagtcgatttatgctccatcacaacgtctct**ctcgc**tgtcg |
| **6** | t**ctcgc**tttctccctccctgctgctggcctcagc**ctcgc**ctctctctcctcttactctcccagctccgcttccacctcccggcctcttgcggcggcctagct |
| **7** | aagcaagct**ctcgc**tagcggcgcccggccattatatatagctcag**ctcgc**ctgcaactgccaatctcaggcatcaaatctagctataa |
| **8** | tgaacgaataattaaccgtaaatgtgaacacccatatcaaatttaagatttgaacctaaacctgctccgctcatttcgtgaaattaattaatttcgttaagaaggaaaaaaaggaaaaaggaagaagaaaagtacatacttgatgcg |
| **9** | gtcgtcgtcgaggacgtcgatctcggaggaccagatcgccgagcttctctccaagcttcaggccctgctcccggagtctcagg**ctcgc**aatggcgcccatagggtaagcatagca |
| **10** | ccagattgcttttatatccattagcccacatgcaagcaagagctaaccaaagatgagtctcatgttcatcttggccaacaatttcttactttctttctctacagtactagcagactaatt |
| **11** | taatcaatacattaaaatcaatcgaactagtattataattaaaaatcgttttcagttgacccggcaggccgggggatcgatcgagagcgaattaaggatcagattttatgagcacgatcgatcacgatt |
| **12** | tcatcataacgatgccagcagtcgcttgcaacatgcc**ctcgc**ttgtgacacacttggcacacggggcgagtatcattgc**ctcgc**ccgcgtccctgacttgcgccgctaggggcgccgcggccgtgaccgccacgaccgaagccgccgcgtccgccacctctgtttccagtgctgctgccg |
| **13** | acacctccactccttcagacaggtgcagccaagatagatagctgaagggagaaaaggaaacacagcagttgcttctagttattaactactagcaagacaacttcgctacactgatcgata |
| **14** | gagatttggaggccaaacagagggttggagattgccgccgccatagaagag**ctcgc**catgtcgctgggagaaagtttgcacgacagaactaatctaatctagcgggaggctctgatacca |
| **15** | tattatatgtgagaaatatctaacactactccacactgtccacaattactgcgaacccgcaacgcaccaaaagcccctacgcgcca**ctcgc**agtttagcacgactacgagatgagagcac |
| **16** | ggtccgaaggtggggacgccaccaccaccagtagaatctagccaatccagttgcaataaagtatgcccctcccgtaccctcctcgtgag**ctcgc**ccactgcgtccccaccacccatacac |
| **17** | ccctctcattagtcaatactgtagctgtagtagtggtagcacccaacac**ctcgc**cattaaagaaacttgag**ctcgc**caagtggagaaaggagaggtggaggagaggaggggaggggaggggaggtg |
