# Supplementary material for: Dynamics of Brassinosteroid Response Modulated by Negative Regulator LIC in Rice
Source: PLoS Genet. 2012 Apr 26;8(4):e1002686. doi: 10.1371/journal.pgen.1002686 (PMC3343102; doi:10.1371/journal.pgen.1002686)
Supplement: Table S4 — Primers used in this study. (DOC) [file pgen.1002686.s015.doc]

**Supplemental Table S4. Primers used in this study.**

| Primers | Sequences |
| --- | --- |
| RP | 5' CTGGACAGAGGAAGCAGGAG 3' |
| LB | 5’ AAT CCA GAT CCC CCG AAT TA 3’ |
| P1/LP | 5’ GAGTCGGCGGCAGGAGATT 3’ |
| P2 | 5' TTAGTTCCTCGAAGCTAAT 3' |
| P3 | 5' GAGGTGGTTCAACCAGCGG 3' |
| LIC-OX F | 5’ CTCGAGATGAGTCGGCGGCAGGAGA 3’ |
| LIC-OX R | 5’ GGTACCAAACACATGGCTAACGTGC 3’ |
| LIC-N F | 5’ CAGCGCGGAAGTTGCAAGTAC 3’ |
| LIC-N R | 5’ GTCCAATATTAGTTGCTGCTC 3’ |
| LIC-AD F | 5’ 'GAATTCATGAGTAGGAGGCAGGAGA 3’ |
| LIC-AD R | 5’ GGATCCCTTAAAACACATGGCTAACGT3’ |
| LIC-realtime F | 5’ ATGCAGACAACAAATTTC 3’ |
| LIC-realtime R | 5’ GCAACTAATATTGGACCGCTA 3’ |
| LICm F | 5’ CTCGAGATGAGTCGGCGGCAGGAGA 3’ |
| LICm R | 5’ GGTACCGGTGCAGAGCTTGGATG |
| LICp F | 5’ CTCGAGATGAGTCGGCGGCAGGAGA 3’ |
| LICp R | 5’ GGTACCTGCCCACATTCATTTAAAT 3’ |
| GSK1 F | 5’ CCCGGGGATGGAGGCGCCGCCGGGGCC 3’ |
| GSK1 R | 5’ GTCGACGTTACGTCCCAGCATGCGCA 3’ |
| SKETHA F | 5’ CCCGGGGATGGCCGCGATGCCAGGTGG 3’ |
| SKETHA R | 5’ CTGCAGGTTAAAATCCTGAATGCCGCT 3’ |
| BIN2 F | 5’ GAATTCATGGCTGATGATAAGGAGAT 3’ |
| BIN2 R | 5’ GGATCCCTTAAGTTCCAGATTGACCTA 3' |
| BZR1-A1 F | 5’ GCGCCACTCGCAGTTTAG 3’ |
| BZR1-A1 R | 5’ TACCCACCAGCTCCGTCC 3’ |
| BZR1-A2 F | 5’ TCCCTTGATAGAGAGGAG 3’ |
| BZR1-A2 R | 5’ GATAAGAGTTGTTGGGC 3’ |
| BZR1-A3 F | 5’ TTACTAAGACAAGGCAAT 3’ |
| BZR1-A3 R | 5’CCGCGGCGCCCCTAGCGG 3’ |
| ILI1-B1 F | 5’ TAGCTTGCTCTTCTAGCT 3’ |
| ILI1-B1 R | 5’ CACGTCTCCTGCAAAACC 3’ |
| ILI1-B2 F | 5’ CAGAGTACAGACAAATCC 3’ |
| ILI1-B2 R | 5’ TAGGTGTGCGTAGCTTAT 3’ |
| ILI1-B3 F | 5’ AGATTTTGGAAACAAAAAG 3’ |
| ILI1-B3 R | 5’ GAGCTACTAGCTGCAGC 3’ |
| IBH1-C1 F | 5’ CATTAGCCCACATGCAAGC3’ |
| IBH1-C1 R | 5’ GAAGTGGAAGGCCAGCAT 3’ |
| IBH1-C2 F | 5’ GGCATTGGAATATTTTTT 3’ |
| IBH1-C2 R | 5’ TCGATTTGTTGTTAAATTG 3’ |
| IBH1-C3 F | 5’ ATATTTATTACTCACTGT 3’ |
| IBH1-C3 R | 5’ TCGATTTGTTGTTAAATTG 3’ |
| Actin1-F | 5’ TGCTATGTACGTCGCCATCCAG 3’ |
| Actin1-R | 5’ AATGAGTAACCACGCTCCGTCA 3’ |
